# Blind and endmember guided autoencoder model for unmixing the absorbance spectra of phytoplankton pigments

**DOI:** 10.1038/s41598-025-96023-5

**Published:** 2025-04-16

**Authors:** Pritish Naik, Ilkka Pölönen, Pauliina Salmi

**Affiliations:** Faculty of Information Technology, University of Jyväskylä, Jyväskylä, Finland

**Keywords:** Spectrophotometry, Computational science, Environmental sciences, Computational biology and bioinformatics, Computational models, Machine learning

## Abstract

Hyperspectral sensing of phytoplankton, free-living microscopic photosynthetic organisms, offers a comprehensive and scalable method for assessing water quality and monitoring changes in aquatic ecosystems. However, unmixing the intrinsic optical properties of phytoplankton from hyperspectral data is a complex challenge. This research addresses the problem of non-linear unmixing hyperspectral absorbance data of concentrated water samples using Blind (BAE) and Endmember Guided Autoencoder (EGAE). We show that spectral unmixing using the EGAE model with different objective functions can effectively estimate the abundance of different optical components in spectral data. The EGAE model demonstrated a higher correlation between unmixed endmember abundances and ground truth for chlorophyll-a (chl-a) and fucoxanthin (fx) biomarker pigment concentrations compared to the BAE model, effectively unmixed the absorbance spectrum of cyanobacterial pigment phycocyanin (pc) and was robust to changes in network architecture. It can adaptively unmix various endmembers without impacting the abundance estimates of other pigments. Our results demonstrate that EGAE provided stable abundance estimates and improved the accuracy and reliability of identifying and quantifying pigments, allowing for more precise unmixing of hyperspectral data into their constituent endmembers. We anticipate that our study will serve as a starting point for targeted unmixing of specific photosynthetic pigments using EGAE.

**Supplementary Information:**

The online version contains supplementary material available at 10.1038/s41598-025-96023-5.

## Introduction

Hyperspectral technology is a key solution to comprehensive, real-time environmental monitoring, as hyperspectral instruments can be used at different volumetric scales from laboratory^1,2^ to aerial imaging and observation from Earth’s orbit^3–5^. Hyperspectral imagers are instruments that produce a data cube instead of a two-dimensional picture, where each pixel contains a spectrum of electromagnetic radiation of the imaged target with hundreds of contiguous wavebands^6^.

Phytoplankton, i.e., microscopic, photosynthetic organisms that live freely in the water column, are central targets of monitoring because they are the basis for the global carbon cycle and respond rapidly to environmental changes^7^. Despite being small, phytoplankton can be numerous, occupying the euphotic layer of a water body, which makes them detectable by spectroscopic methods^8^. The composition and concentration of pigments are the visible optical properties of phytoplankton that affect the shapes of their absorbance and reflectance spectra. All freshwater phytoplankton species contain chl-a, but the presence of other chlorophylls, carotenoids, and phycobilins varies^9^. Together with chlorophylls, phycobilins cause the characteristic cyan tint of cyanobacteria. The composition of pigments roughly follows the species composition of phytoplankton, indicating whether the prevailing species could be harmful cyanobacteria or nutritious, carotenoid-rich species^10^ .

Inland and coastal waters are complex matrices of dissolved and suspended material, also other than phytoplankton, affecting light propagation and, therefore, their hyperspectral monitoring. The key challenge with hyperspectral observation, including point-sourced spectroscopy and hyperspectral imaging, is how to unmix the signals from different coloured components when one pixel contains information from several different, usually unknown, sources, also known as endmembers. Researchers have proposed various physics-based and data-driven unmixing models to extract endmembers and estimate their abundances, which are the proportions of each endmember. Non-linear unmixing models focus on the complex physical interactions between light and various materials observed on both macroscopic and microscopic levels. Linear models simplify these interactions by assuming that light only interacts with a single material at a macroscopic level and that any pixel observed is merely a linear mixture of endmembers, weighted by their abundance fractions^11^.

Typically, algorithms are constrained by modeling errors and observational noise resulting in simplifications with low generalizability^11^. Hyperspectral unmixing is a challenging, ill-posed problem that is an attractive task for deep learning techniques, including supervised, semi-supervised, or unsupervised neural networks. The deep autoencoder networks are of particular interest because of their unsupervised or self-supervised nature and their ability to approximate endmembers from training data without needing each pixel to represent a single material. Deep autoencoder networks are used primarily for unsupervised learning of efficient data codings and are specifically designed to encode input data into a compressed, lower-dimensional representation and then decode that representation to produce output that is as close to the original input as possible^12^. A deep autoencoder is a more complex version of a basic autoencoder and consists of two parts: an encoder and a decoder with multiple hidden layers.^13^ proposed a deep autoencoder-based model for linear unmixing that focuses on extracting endmembers and validated the model against a previously established endmember ground truth. The model was applied to the Jasper Ridge dataset to extract endmembers such as roads, soil, water, and trees, and then these were compared with ground truth data.^14^ introduced a non-linear unmixing model to unmix four pure material endmembers from the synthetic data of the USGS digital spectral library and six endmembers, namely asphalt, grass, tree, roof, metal, and dirt, from real data in the Urban dataset.

A non-linear, deep autoencoder could be particularly suitable for unmixing components of suspensions, assuming complex interactions among light, substances, and the suspension medium itself.^15^ proposed a blind convolutional deep autoencoder for unmixing hyperspectral images over water bodies. The autoencoder model showed good unmixing performance on benchmark datasets and NASA’s 2017 HSI2 image of Lake Erie, successfully detecting chl-a and distinguishing various water types. However, deep autoencoder-based unmixing could be expanded to analyze the spectra of phycocyanin, carotenoids, and other photosynthetic pigments in various water bodies.

We aimed to test deep autoencoder networks to find the endmembers corresponding to the spectra of pigments (chl-a, fx, pc) and background endmembers from the absorbance spectra measured from concentrated water samples. The abundances were expected to correlate with measured concentrations of pigments. The dataset used here was absorbance spectra of samples collected from 20 lochs in Scotland, recorded with a hyperspectral imager^2^. Given that the absorbance spectra of pure pigment extracts are well known^16,17^, this study explored the possibility of using these pure pigment spectra as fixed endmembers. Blind spectral unmixing and endmember-guided unmixing approaches were tested, hypothesizing them to perform equally well for both spectral unmixing and reconstruction. Notably, previous research has shown that reconstruction performance does not necessarily imply better unmixing performance^13^. Here, the impact of different loss functions was studied with models optimized for reconstruction error, and ways to control unmixing performance were discussed. Finally, recommendations were made for the use of deep autoencoder networks to solve the ill-posed inverse problem of complex mixtures presented by inland waters.

## Results

**Fig. 1:**
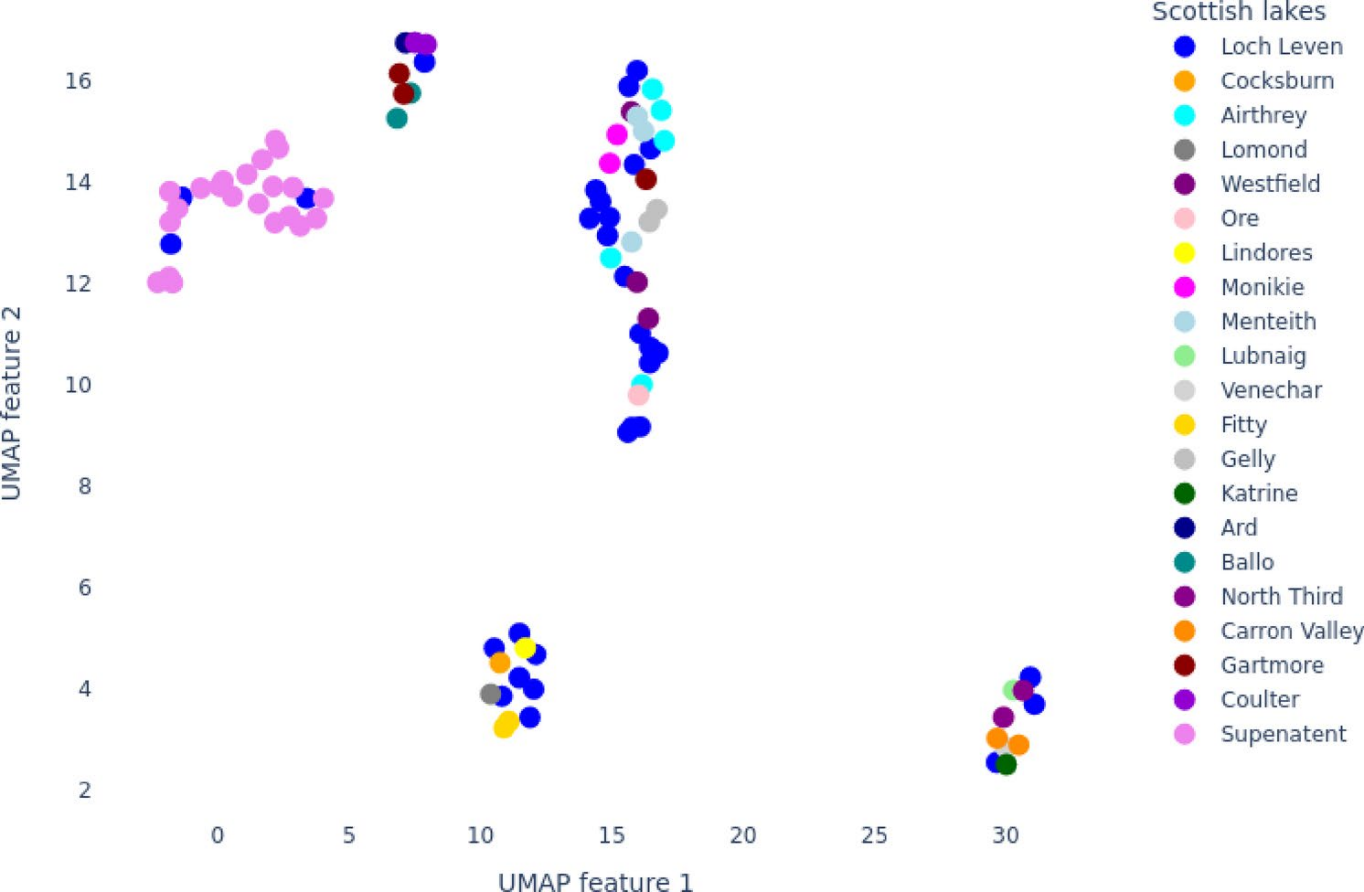
UMAP visualization of mean absorbance spectra of lake water samples. The blue color represents Loch Leven. The Loch Leven samples are dispersed across all the clusters formed with samples from other lakes, indicating that the spectral characteristics of Loch Leven water are diverse and share similarities with those of other lakes in the study.

We split the hyperspectral data of 20 lakes into two categories: Loch Leven as the test set and the remaining 19 lakes as the training set. The rationale behind this categorization was that Loch Leven effectively represents the 19 lakes, as shown in the Uniform Manifold Approximation and Projection (UMAP) plot (Fig. 1).

Our proposed BAE and EGAE autoencoder architectures consisted of an input/output layer, intermediate hidden layers, and a latent layer. However, in EGAE, the latent layer was split into two segments: one connected with non-trainable weights of the first layer of decoder fixed with the pigments endmembers^16,17^ and another with an adjustable number of background endmembers. The number of background endmembers was set as a hyperparameter, allowing the hyperparameter optimization method to determine their optimal count because their exact nature was unknown.

The training dataset was used to optimize the hyperparameters in the BAE and EGAE. The Hyperband Keras Tuner identified the optimal hyperparameters for BAE and EGAE. The best models were defined by the least reconstruction loss. We selected the 10 best BAE models, each having a different set of hyperparameters optimized using MSE (Mean Squared Error) as the loss function. Additionally, we tested BAE models optimized with SAD (Spectral Angle Distance) and SID (Spectral Information Divergence), but these were omitted from further analysis due to low performance (Supplementary Table 1, Supplementary Fig. 1). For the EGAE, we selected the 10 best models for each of the following loss functions: MSE, SAD, SID, and the weighted sum of MSE and SAD. With these hyperparameters fixed, we extracted the abundance estimates for the test set from the latent layer activations by using just the encoder part of the autoencoder.

The results section is organized as follows: (1) We compare the results of the selected BAE and EGAE using MSE loss, selected from the 10 best models according to reconstruction loss, to assess the improved unmixing performance of the EGAE. (2) We analyze unmixing performance across the 10 best models for different network configurations: BAE with MSE loss and EGAE with MSE, SAD, SID, and weighted MSE and SAD loss. (3) We evaluate the stability of the EGAE and BAE models by comparing the variation in abundance estimates across different network configurations.

### Unmixing performance of a selected BAE and EGAE: endmember and abundance estimates

**Fig. 2:**
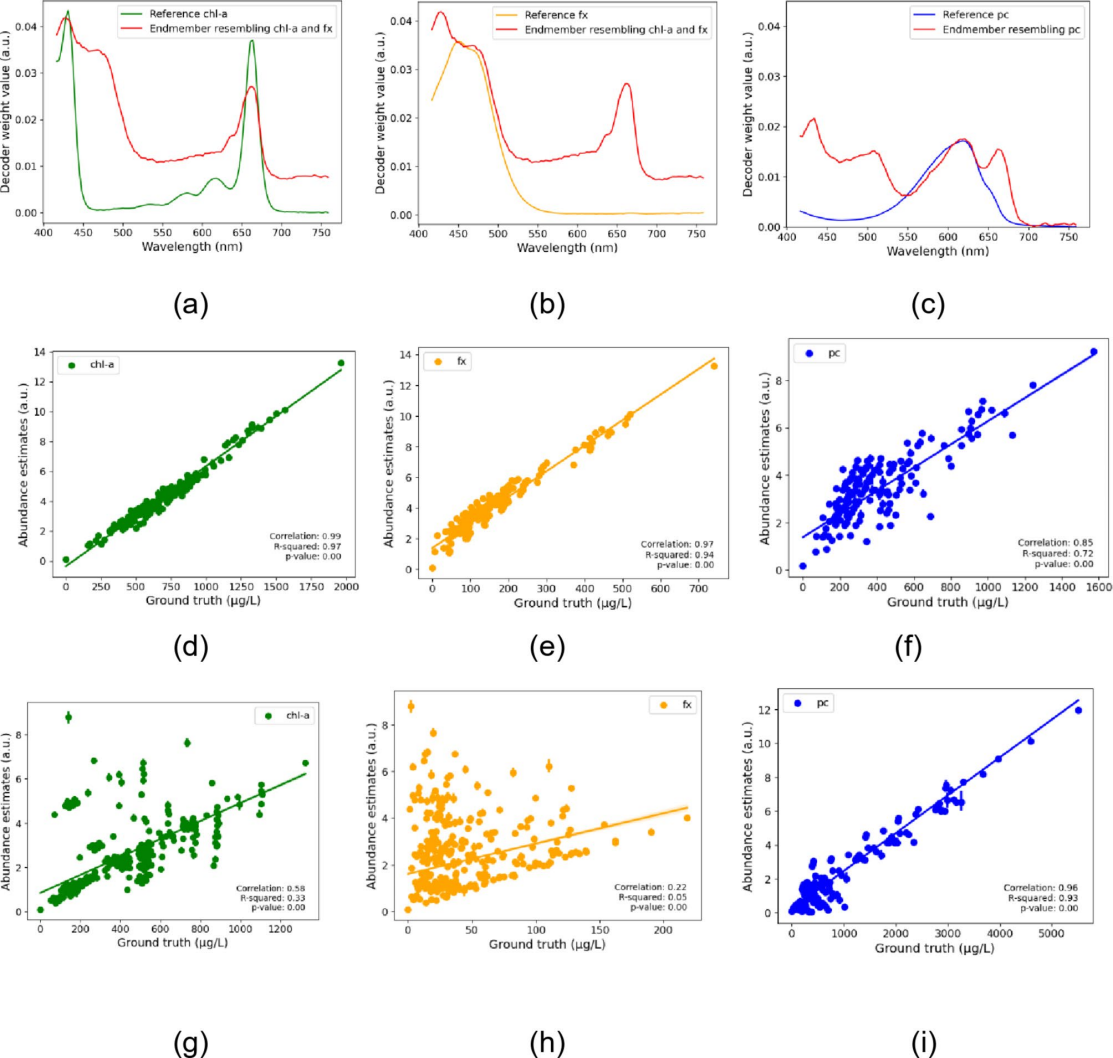
Endmember estimates (**a-c**) and scatter plots between ground truth and abundance estimates for a selected BAE model with MSE loss for the test (**d-f**) and training set (**g-h**). The abbreviations chl-a, fx, and pc stand for chlorophyll-a, fucoxanthin, and phycocyanin respectively and correlation refers to Pearson correlation. All the correlations are statistically significant $\:(p<0.001)$.

In the blind unmixing model (BAE), the exact pigment spectra cannot be confirmed because the extracted endmembers are approximations, not pure spectra. The endmembers extracted from the BAE model for the three pigments did not exactly match the reference absorbance spectra of the pigments in acetone^16,17^, likely due to inadequate unmixing or masking by other pigments^7,18^. Therefore, we applied a multi-step process to match endmembers with reference pigment spectra, using the Spectral Angle Distance (SAD) metric for comparison and visually confirming that the extracted endmember contained the peak of the pigment spectra. Together with SAD values, visual matching of peaks between the extracted and reference spectra indicated successful unmixing by the model. Figures 2a-c show the endmembers resembling chl-a, fx, and pc from the BAE model compared with reference spectra. The chl-a and fx reference spectra^16^ matched with the same endmember estimate, with overlapping peaks, indicating they were not completely unmixed from each other (Fig. 2a and b). The endmember resembling pc matched well with the pc reference peak^17^ but also contained other components (Fig. 2c).

Based on matched endmembers, we took the corresponding abundance estimates and compared them with ground truth concentrations for the training and test sets of a selected BAE model with MSE loss. The results show positive Pearson correlations for chl-a, fx, and pc, all of which are statistically significant $\:(p<0.001)$. Scatter plots show a strong positive correlation for chl-a (Fig. 2d g) and pc (Fig. 2f and i) in the training and test set. For fx, the correlation was strong in the test but weak in the training set (Fig. 2e h).

**Fig. 3:**
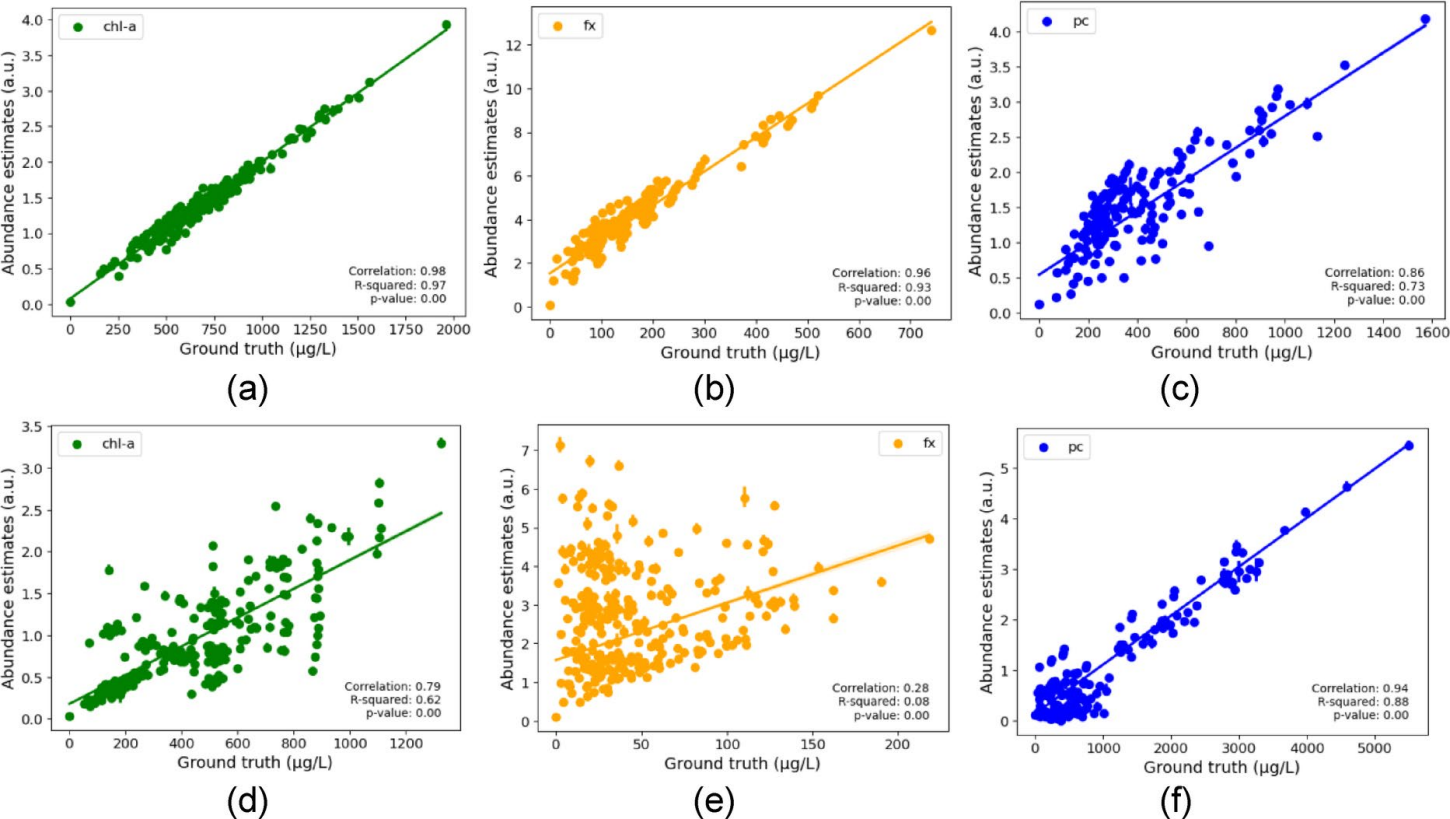
Scatter plots between ground truth and abundance estimates for a selected EGAE model with MSE loss for the test (**a-c**) and training set (**d-f**). The abbreviations chl-a, fx, and pc stand for chlorophyll-a, fucoxanthin, and phycocyanin, respectively, and correlation refers to Pearson correlation. All the correlations are statistically significant $\:(p<0.001)$.

In the EGAE, we had fixed endmembers for the pigments, so we obtained corresponding estimates without the need for spectra matching with reference as in the BAE model. The scatterplot illustrating the Pearson correlation between the ground truth concentration for chl-a, fx, and pc, and abundance estimates across training and test sets for one of the best EGAE models with MSE loss is shown in Fig. 3. Figures 3(a) and 3(d) show strong correlations for chl-a (test and training), 3(b) and 3(e) show strong (test) and weak (training) correlations for fx, and 3(h) and 3(i) show strong correlations for pc (test and training). The non-augmented test data results were consistent with augmented test data (Supplementary Fig. 2).

In summary, the BAE model’s correlation for chl-a was close to that of the EGAE models. However, for fx, the BAE model lagged in both training and test sets and failed to unmix pc, while EGAE successfully unmixed pc with strong correlations to the ground truth, showcasing the better performance of EGAE in pigment identification. This emphasized the need for strong correlations of abundance estimates with ground truth and close resemblance of the corresponding endmember to pure pigment for controlled pigment identification, both of which were ensured by EGAE.

### Ground truth and endmember abundance correlations and unmixing performance of 10 best models for BAE and EGAE with different loss functions

The mean Pearson correlation for the training and test sets between abundance estimates and the ground truth of chl-a, fx, and pc across the ten best models for BAE and EGAE with different loss functions identified by hyperparameter optimization, assessing the consistency of unmixing performance, is shown in Table 1.

**Table 1:** Pearson correlation and coefficient of variation for the 10 best models with 5 different configurations (BAE-MSE, EGAE-MSE, EGAE-SAS, EGAE-SID, EGAE-weighted MSE-SAD) for test and training set. The best models were chosen using the hyperband hyperparameter optimization method. The correlation value is the mean pearson correlation of the 10 best models for each configuration. Stability is indicated by the coefficient of variation of the linear regression line for these models. All correlation coefficients were significant. Bold highlights indicate the best performance, and hyphen (-) indicates that no values are reported due to poor unmixing. (*p* < 0.001).

Dataset	Model	Pearson correlation			Coefficient of Variation (%)			Spearman correlation		
		chl-a	fx	pc	chl-a	fx	pc	chl-a	fx	pc
Test set (Loch Leven)	BAE – MSE	0.98	0.93	-	15.08	59.80	-	0.98	0.87	-
	EGAE – MSE	**0.99**	0.96	**0.85**	**6.35**	**7.54**	**24.65**	**0.98**	0.94	**0.78**
	EGAE – SAD	0.98	0.94	0.84	103.34	124.70	138.71	0.97	0.90	0.73
	EGAE – SID	0.97	0.94	0.82	111.99	88.05	133.19	0.97	0.90	0.72
	EGAE – MSE - SAD	0.98	**0.97**	0.77	9.13	9.03	35.67	0.97	**0.94**	0.74
Training set (19 lakes)	BAE – MSE	0.77	0.29	-	17.40	53.70	-	**0.84**	0.53	-
	EGAE – MSE	**0.78**	0.30	0.95	7.82	**7.92**	15.17	0.82	0.47	0.70
	EGAE – SAD	0.74	**0.46**	0.73	106.52	112.34	128.82	0.80	**0.54**	**0.78**
	EGAE – SID	0.75	0.46	0.70	114.95	85.58	126.28	0.80	0.54	0.75
	EGAE – MSE - SAD	0.78	0.27	**0.95**	**5.09**	12.38	**9.34**	0.82	0.45	0.54

With MSE loss, the best 10 BAE models successfully unmixed two of three pigments but failed to unmix all three pigments simultaneously (Table 2). Specifically, the BAE model was able to unmix the absorbance spectrum of chl-a, fx but the results for pc were unsatisfactory because pc was often mixed with either chl-a or fx (Table 2) (Fig. 2i). We say that chl‑a and fx were unmixed in BAE because their distinct peaks are clearly visible in the extracted endmember (Fig. 2i). However, the pc peak around 620 nm overlaps with the chl‑a feature, making it difficult to confirm its unmixing. Only two times out of the ten best models chosen using hyperparameter optimization, were we able to see pc peak contained in the extracted endmember.

**Table 2:** The similarity between BAE - MSE endmembers resembling reference endmembers in Rad in the 10 best models from the training set. Mean SAD is the mean spectral angle distance between the unmixed endmembers and absorbance spectra derived from^16,17^ the asterisk(*) highlight shows that the SAD values of the reference spectra were compared with the same endmember, indicating incomplete unmixing.

	Models										Mean SAD
	1	2	3	4	5	6	7	8	9	10	
chl- a	0.76*	0.73*	0.76*	0.75*	0.77*	0.77*	0.75*	0.76*	0.77*	0.70*	0.75
fx	0.40	0.31	0.30	0.37	0.50	0.47*	0.45*	0.35	0.35	0.53	0.40
pc	0.76*	0.89*	0.75*	0.71*	0.86*	0.74	0.80	0.90*	0.61*	0.92*	0.79

The EGAE effectively unmixed the three pigment endmembers. The EGAE with MSE loss successfully extracted all three endmembers in 7 out of 10 best models, yielding the most consistent unmixing results. (Table 3)

**Table 3:** Unmixing performance of 10 best EGAE. The unmixing success rate is the number of times all three pigments were unmixed. Columns 3 to 5 show the percentage of times each pigment was unmixed in the 10 best models.

EGAE - Loss Function	Unmixing Success Rate (%)	Endmembers unmixed from the 10 best EGAE models with the least reconstruction error. (%)		
		Chl-a	Fx	Pc
MSE	70	90	70	80
SID	60	90	60	100
SAD	70	90	70	100
MSE-SAD	10	90	70	40

In the training set, all BAE and EGAE models with different loss functions showed similar Pearson correlations for chl-a, with EGAE-MSE and with EGAE weighted MSE-SAD performing slightly better (Table 1). For fx, EGAE using SID and SAD scored higher (*r* = 0.46) than MSE (*r* = 0.30), MSE + SAD (*r* = 0.26), and BAE (*r* = 0.29), showing the effectiveness of scale-invariant loss functions. For pc, EGAE with MSE and MSE + SAD (*r* = 0.95) outperformed SID (*r* = 0.70) and SAD (*r* = 0.73) and even more so BAE, which failed to unmix pc and estimate its abundances (Table 1).

In the test set, the EGAE with MSE loss showed the strongest correlations for chl-a (*r* = 0.99) and pc (*r* = 0.85) and ranked second for fx (*r* = 0.96), just below the EGAE with weighted MSE-SAD. The EGAE with weighted MSE-SAD loss achieved the highest correlation for fx at *r* = 0.96, exceeding the BAE model’s *r* = 0.93. This demonstrated the strong unmixing performance of EGAE for the three pigment endmembers (Table 1).

To demonstrate robustness and generalization, we used a leave-one-lake-out approach across 19 lakes, training on 19 (including loch Leven) and testing on the remaining one. This produced 19 independent evaluations with Pearson correlations of 0.60 (chl-a), 0.38 (fx), and 0.66 (pc) (Supplementary Fig. 5). We also tested EGAE with MSE loss across four SNR levels, demonstrating strong performance even in high-noise scenarios (Supplementary Table 2, Supplementary Figs. 3 and 4).

### Stability analysis across the 10 best models for BAE and EGAE with different network configurations and loss functions

Our analysis of ground truth pigment concentrations versus abundance estimates showed significant variability across different BAE model configurations and executions of the same topology. This inconsistency could be attributed to inadequate endmember unmixing, over-parameterization, or variations in model architecture, such as the number of endmembers, layers, and units.

Variation in abundance estimates across 10 models for each of the 5 configurations (BAE-MSE, EGAE-MSE, EGAE-SAD, EGAE-SID, EGAE-weighted MSE-SAD) was analyzed. The stability was analyzed using the coefficient of variation for the linear regression line for scatter plots between ground truth concentrations and abundance estimates. A lower coefficient of variation indicates more stable abundance estimates across 10 best models (Table 1). BAE models (MSE loss) showed high coefficients of variation in the test and training set (Table 1). EGAE models significantly improved stability, especially with MSE loss and weighted MSE - SAD loss (Table 1).

**Fig. 4:**
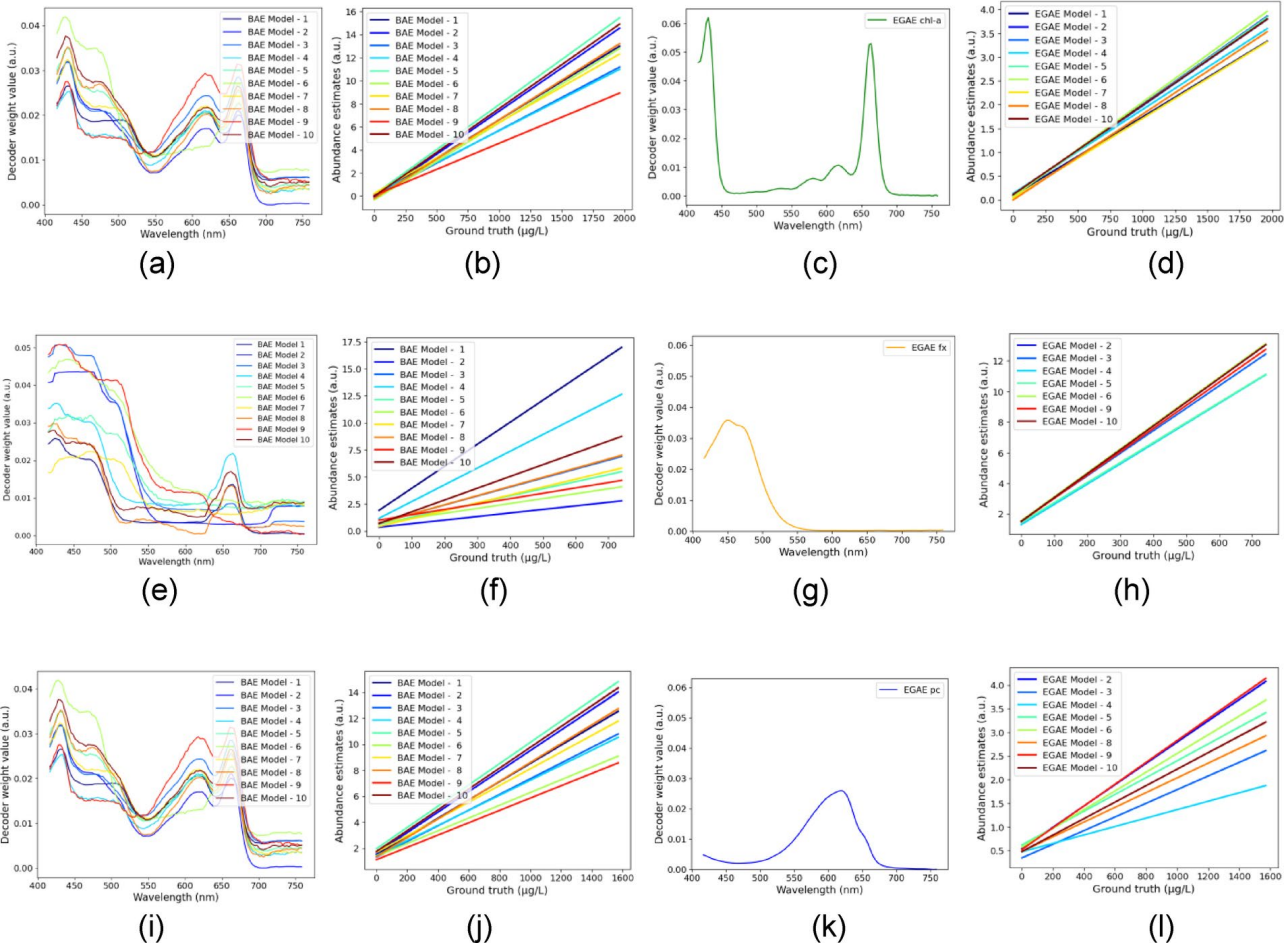
Improved stability of abundance estimates for pigment endmembers using the 10 best EGAE models (MSE loss) compared to the 10 best BAE models (MSE loss) for test set. (**a**) (**e**) and (**i**) show unmixed endmembers resembling the chl-a and fx spectra from the literature using the BAE models. (**c**) (**g**) and (**k**) show fixed endmembers using the reference spectra for chl-a, fx and pc for the EGAE models. (**b**), (**f**) and (**j**) show regression lines of scatter plots of ground truth and abundance estimates in BAE models, showing considerable variation, while (**d**) (**h**) and (**l**) demonstrate enhanced stability with EGAE models.

Figure 4 shows improved stability of abundance estimates for the pigment endmembers using the 10 best EGAE models (MSE loss), compared to the 10 best BAE models (MSE loss). As shown in Fig. 4b and f, the BAE model shows a high spread of regression line slopes, indicating low stability in the abundance estimates for pigments chl-a and fx. Conversely, Fig. 4d, 4 h show that the EGAE has a relatively lower spread of regression line slopes, indicating high stability in the abundance estimates. Although Fig. 4j suggests the BAE model has a lower spread for pc compared to EGAE’s Fig. 4l, this is misleading because BAE failed to unmix pc, often appeared mixed with chl-a or fx. Therefore, the EGAE was more stable in estimating all three pigments.

## Discussion

In aquatic environments, there is a high complexity in hyperspectral unmixing arising from intricate interactions between pure endmembers, resulting in challenges that may not be effectively solved by linear unmixing models. Our study presents an efficient computational method for non-linear unmixing of color components corresponding to spectral signatures of pigments (chl-a, fx, and pc) and background absorbance spectra from water samples and considering the complex light-water interactions. The EGAE can map the composition of phytoplankton communities into lower dimensional latent space that contains sufficient information to enable the study of key characteristics and functionality, such as pigment concentrations and taxonomic diversity. This implies that high-dimensional hyperspectral data of water samples encapsulate low-dimensional latent information that the deep learning model can utilize for enhanced phytoplankton monitoring.

The ground truth concentrations of pigments (chl-a, fx, and pc) strongly correlated with EGAE abundance estimates in the Loch Leven dataset, as hypothesized. The natural variations in 19 lakes due to phytoplankton pigment combinations and other constituents in water, such as non-algae particles and dissolved organic matter, led to variability in the spectral data in the training set, resulting in poorer correlations than those observed in the single-lake Loch Leven test set. Please note that the x- and y-scales for the training set differ from those of the test set, and the error is higher at lower concentrations, closer to the detection limits of the analytical method. In the training dataset of 19 lakes, chl-a and pc showed strong positive correlations, while fx improved from weak positive correlations with scale-variant loss (MSE) functions to moderate correlations with scale-invariant loss functions (SID, SAD), supporting the hypothesis that loss function choice affects endmember unmixing performance in deep autoencoder models. Contrary to expectations, EGAE consistently outperformed BAE in extracting endmembers and correlating actual pigment concentrations with estimated abundances. This study supports that a deep autoencoder model with a data prior (EGAE) could be the preferred strategy for unmixing primary pigments like chl-a for accurate abundance estimations and could be extended for secondary pigments like fx and pc, given their expected presence in water samples.

^13^ introduced a blind linear unmixing model using an autoencoder neural network with deep and shallow encoders and a linear decoder. They tested configurations involving different objective and activation functions, with each run iterated 50 times, using the Vertex Component Analysis for initialization of decoder weights. The model was evaluated on three airborne hyperspectral imaging datasets - Samson, Jasper Ridge, and Urban - featuring 3, 4, and 6 endmembers, respectively. The extracted endmembers were compared against ground truths using the SAD, achieving mean values of 0.031, 0.078, and 0.151. Our blind unmixing model (BAE), focusing on water samples and pigments like chl-a and fx, yielded mean SAD values of 0.7 and 0.39 using MSE as the loss function. Although direct comparisons are challenging due to dataset differences and SAD values not being directly comparable, their findings concluded that the choice of autoencoder configuration and loss function is dataset dependent. Our research with the EGAE proposes that, in addition to dataset dependence, the choice is also endmember-specific, with scale-invariant functions outperforming scale-variant ones for fx in our study.

^14^ introduced a non-linear unmixing model using an autoencoder with a post-nonlinear decoder, applying Root Mean Squared Error for assessing abundance estimation accuracy and SID and SAD to evaluate the similarity between true and estimated endmembers. Their study processed two datasets generated via linear, non-linear, and bilinear mixing models, using endmember signatures from the USGS digital spectral library and an airborne HSI—Urban dataset, comparing actual ground truth abundance values at each pixel with the model’s estimates. In contrast, our study utilized real datasets from water samples instead of standard airborne HSI or synthetic datasets. We propose an advanced unmixing model using an autoencoder with a post-nonlinear additive decoder and a two-pass approach: first, estimating abundances and endmembers for training data, then extracting the encoder in the second pass to estimate abundances for the Loch Leven test set. We validated our results by correlating the estimated endmember abundances with concentrations of pigments measured in laboratory assessments.

Recently, the NSAE-SU (non-symmetrical autoencoder for spectral unmixing) model was introduced for spectral unmixing to perform endmember extraction and fractional abundance estimation^15^. This model was evaluated using Jasper Ridge and Samson benchmark datasets and the HSI2 dataset acquired by NASA over Lake Erie in 2017. The extracted endmembers from reflectance data were compared with reference endmember signatures using the SAD metric. For the HSI2 image, the best match for the red Region of Interest was the Chl-a-3 spectrum, with an SAD value of 0.369. Our input was absorbance data, and the blind unmixing model (BAE) produced multiple endmembers, some with SAD values close to the reference endmember difficult to choose the best match. It was observed that endmembers generated from the BAE model may not necessarily match the reference endmembers, because the SAD metric evaluates the spectral shape, not considering other factors that might better match the spectra’s characteristics. For instance, a background endmember resembling chl-a might yield a better SAD value than the actual chl-a endmember, which could be masked by secondary pigments. This issue was observed in the BAE, which used MSE as the loss function and produced two endmembers resembling fx with very close SAD values (± 0.01). However, when we analyzed the endmember abundance estimates’ correlation with the ground truth concentration, only one provided a strong correlation. Therefore, a favorable SAD value may not necessarily indicate correct endmember identification; the correlation of abundance estimates with actual ground truth concentrations should also be evaluated.

Under ideal conditions, a linear mixing model can effectively unmix mixed pixels in hyperspectral data. However, variations induced for example due to illumination changes, topography, environmental noise, and atmospheric effects result in non-linear mixing, complicating the accurate unmixing of endmembers and abundance estimates. The interest in non-linear blind unmixing has increased with advancements in unsupervised deep learning methods, but various research efforts yield physically meaningless and potentially misleading endmember estimates. This problem becomes more pronounced in scenarios involving covarying components, such as algae pigments, or additional mediums like water bodies alongside air, increasing the complexity of the unmixing process. Therefore, including well-established endmember guidance can achieve reliable abundance and endmember estimates. Autoencoder models used for blind unmixing focus on minimizing reconstruction error, i.e., reducing the difference between input and output data. However, this approach can produce endmembers that lack physical interpretability. Therefore, it is essential to evaluate model performance both in terms of unmixing performance, i.e., how accurately the target endmembers are unmixed, and reconstruction performance.

The EGAE demonstrated high effectiveness in achieving reliable endmember and abundance estimates by using the reference spectral signatures of pigments (chl-a, fx, pc) from the reference^16,17^ and laboratory assessments^2^ to validate abundance estimates against known concentrations. This comprehensive approach inherently improves unmixing performance along with reconstruction performance and enables targeted endmember unmixing of the pigments. Chl-a, the primary pigment, was confirmed to be present in all water samples, which allowed us to accurately unmix it using appropriately adjusted weights of the decoder using reference spectra^16^. The chl-a’s endmember estimate was subsequently used to adjust the decoder weights of endmembers for secondary pigments fx^16^ and pc^17^, thus improving abundance estimates of secondary pigments. Therefore, this endmember-guided approach led to improved unmixing performance over the blind unmixing model (BAE), producing a stronger correlation between ground truth concentrations and abundance estimates for the pigments.

By using the EGAE and analyzing the endmembers and abundances produced by the model, researchers can gain valuable insights into the composition and concentration of different color components within the aquatic environments. This capability allows for more informed decisions concerning aquatic environments. This study was based on HSI data from water samples analyzed using a mobile imager in a laboratory setting. The next phase of this research is to apply the EGAE to hyperspectral imaging (HSI) data obtained from remote sensing and perform targeted unmixing of endmembers for real-time monitoring of endmembers.

## Methodology

### Sampling sites, sample concentration, and spectral imaging

**Fig. 5:**
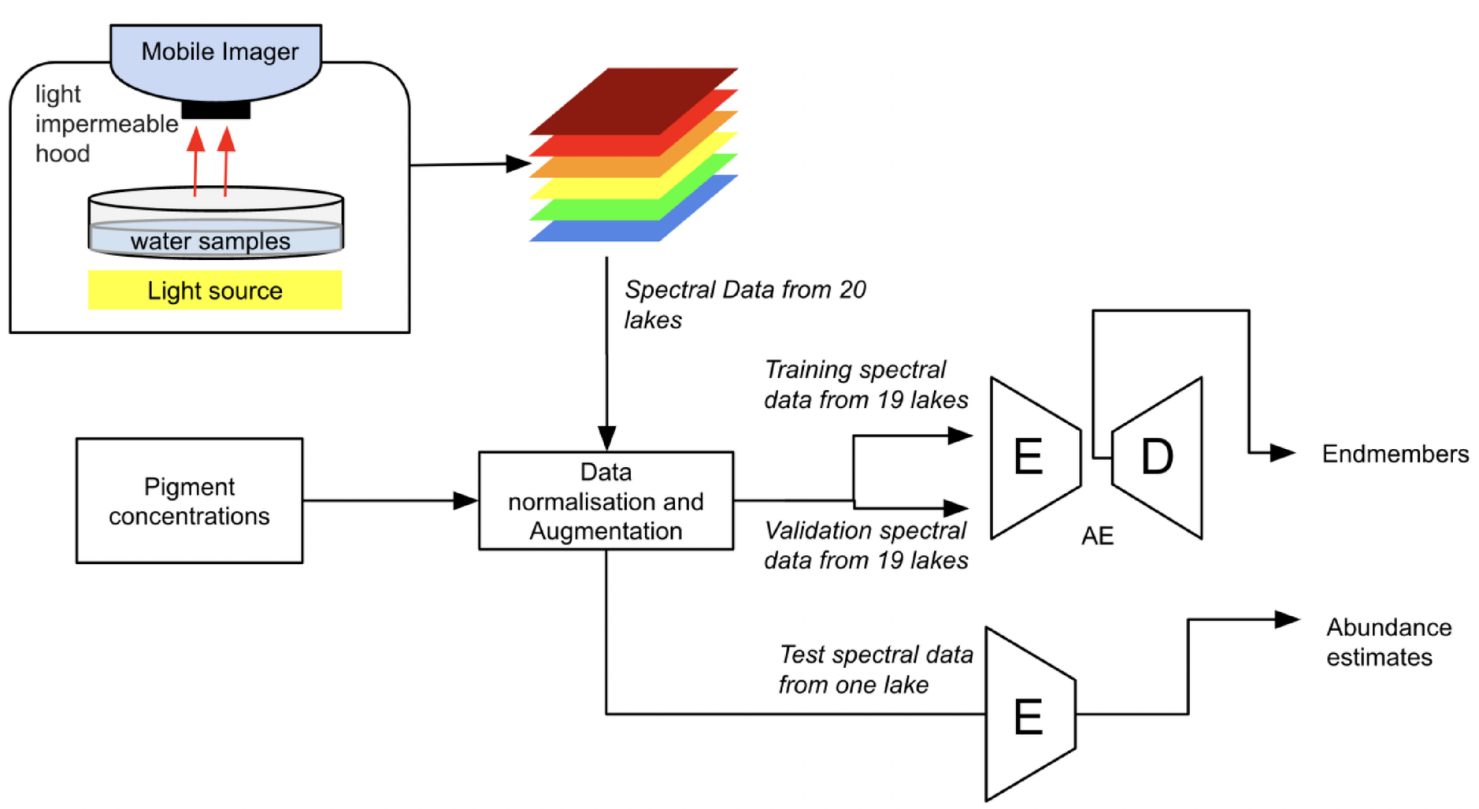
Flow diagram of the transmittance imaging setup and endmember and abundance estimation using either the BAE or EGAE model. Abbreviations: E (Encoder) and D (Decoder) AE (Autoencoder).

The dataset used in this study is described by^2^ and openly available via^19,20^. Water samples were collected from 20 Scottish lochs between May and June 2022. Chlorophylls and carotenoids were assessed using HPLC, and phycocyanin concentration was analyzed using a spectrophotometer (protocol “E” of^21^). The spectra were recorded with a spectral imager from lake water samples concentrated by centrifugation. We used Beer-Lambert approach to convert measured transmittance to absorbance, an approximation chosen due to the complexity of natural water samples, unknown endmembers, and uncertainties in ground truth analyses (Fig. 5). Detailed protocols are provided^2^.

### Data set curation and preprocessing

As illustrated in Fig. 1, the distribution of the Loch Leven set closely resembled that of the 19 lochs, as demonstrated by the UMAP plot. This similarity supported the decision to use the Loch Leven set as test data while training the model on data from the 19 lochs. The idea for this division was to assess whether the model, trained on a diverse set of lakes, could effectively unmix endmembers in samples from a lake that was isolated, independent, and not included in the training dataset. This approach aimed to evaluate the model’s capability to handle the unpredictability and variability of unseen real-world environments.

The training and test datasets consist of spectral images and corresponding ground truth concentrations for chl-a, fx, and pc. Spectral images from 89 samples, each with 120 bands (416 nm to 758 nm), were split into a training set (57 × 120) and a test set (32 × 120), along with the corresponding pigment concentrations for chl-a, fx, and pc for the training set (57 × 3) and test set (32 × 3). Each set included the mean absorbance spectra of 50 × 50 regions of interest from samples of Scottish lakes, with the training set including samples from 19 lakes and the test set from Loch Leven.

Supernatant samples, with pigment concentrations recorded as zero, were included in both the training and test datasets. A spectral mixture model was created by randomly averaging two mean spectra and corresponding ground truth for both sets. The training set contained $\:{X}_{train}$ spectra and corresponding simulated ground truth $\:{M}_{train}$ with 10,000 samples, while the test set contained $\:{X}_{test}$ spectra and ground truth $\:{M}_{test}$with 5,000 samples. The term ‘ground truth’ is used to avoid repetitive mentions of simulation. Therefore, the ground truth refers to simulated ground truth data. This data augmentation method utilized the additive nature of absorbance, retaining the same sources of variation while generating new combinations, some of which might not be ecologically feasible^2^. The augmented absorbance spectra of the training and test data were min-max normalized using the entire dataset.

### Network architecture

#### Notations

Input spectra $\:X\in\:{\mathbb{R}}^{B\times\:N}$ has $\:N$ samples, and $\:B$ spectral bands. The spectra of the sample $\:p$ where $\:p\:=\:1,\dots\:,N$ is denoted by $\:{x}_{p}$. The number of endmembers is denoted by $\:R$. The endmembers are denoted by $\:{m}_{r}$ where $\:r\:=\:1,.R$. and the matrix having the endmembers as columns is denoted by $\:M$ $\:\in\:{\mathbb{R}}^{B\times\:R}$. The abundance vector for sample p is denoted by $\:{s}_{p}=[{s}_{1,p},\dots\:.,{s}_{R,p}]$ and the matrix of all abundance vectors as columns is denoted by $\:S$ $\:\in\:{\mathbb{R}}^{R\times\:P}$.

#### Problem formulation

An autoencoder is a neural network trained to reconstruct its input and prevent it from learning the identity function. Studies^13,14^ observed direct correspondence between the weights of the linear part decoder in BAE and the endmembers matrix, as indicated by the post-non-linear additive unmixing. The observed spectrum of the sample is represented by

1 $$\:y\:=\:MS+\:\rho\:(M,S)\:+\:n,$$

where $\:M$ is the endmember matrix, and $\:S$ is the abundance vector representing the proportion of each endmember. The additive non-linear interaction term between the endmembers is represented by $\:\rho\:(M,S)$. The vector $\:n$ represents the noise in the observed spectrum.

## Proposed solution

The BAE autoencoder model was a fully connected encoder and decoder, each optimized through methods detailed in the hyperparameter selection section. The encoder used multiple ReLU-activated dense layers, with adjustable layer and node counts, aiming for a latent representation based on the number of endmembers. This number was a hyperparameter chosen to minimize reconstruction error. The encoder output abundance values that indicated the proportion of each endmember present in the input. The decoder, reconstructing the hyperspectral data, included a linear section for linear unmixing with non-negative constraints and total variation regularization alongside a non-linear section with two dense layers employing L2 regularization and Leaky ReLU activations. The outputs from the linear and non-linear sections were combined to produce the output spectra, with the entire model optimized using an Adam optimizer with an adjustable learning rate.

The EGAE was built upon the BAE structure with modifications in the latent and decoder layers. It retained the fully connected architecture and fine-tuning approach. The encoder’s design with ReLU-activated dense layers allowed for adaptable configurations. Its latent layer was split into two segments, with one segment connected to the subsequent layer with frozen weights fixed using the literature spectral signatures of pigments, i.e., chl-a, fx^16^, and pc^17^, and another segment connected to the subsequent layer with trainable weights for background endmembers. These segments represented the linear component of the unmixing model, followed by two additional fully connected layers responsible for non-linear unmixing. The reconstructed spectra result from merging the linear and non-linear outputs. The EGAE also employed an Adam optimizer with a hyperparameter adjustable learning rate.

The encoder transforms the input data $\:x\in\:\:{\mathbb{R}}^{N\times\:B}$, where $\:N$ is the number of absorbance spectra and $\:B$ is the number of spectral bands, into the latent representation $\:\text{z}\in\:{\mathbb{R}}^{N\times\:R}$ where $\:R$ is the number of units in the latent layer. The encoder generates a latent representation $\:z$ as $\:z\:=\:{f}_{e}\left(x\right)$ with $\:{f}_{e}:\:\:{\mathbb{R}}^{B}\:\to\:\:{\mathbb{R}}^{R}$. The decoder uncompresses this latent representation $\:z\:$to reconstruct the original input data $\:\widehat{x}\:=\:{f}_{d}\left(z\right)$ with $\:{f}_{d}:\:\:{\mathbb{R}}^{R}\:\to\:\:{\mathbb{R}}^{B}$. The network optimizes the parameters and encodings by reducing the mean reconstruction error between the input $\:{x}_{i}$ and its reconstructed value $\:{\widehat{x}}_{i}=\:{f}_{d}\left({f}_{e}\left({x}_{i}\right)\right)$.

### Encoder architecture

The encoder is a deep neural network composed of fully connected dense layers. The number of units in the input layer equals the number of spectral bands $\:B$, while the number of units in the latent layer equals the number of endmembers $\:R$. The number of endmembers is a hyperparameter determined by the hyperparameter optimization method. All the activation functions in the encoder are ReLU.

The output of the layer $\:i$ is given by

2 $$\:{h}^{\left(i\right)}=\:{\varnothing}\left({W}_{e}^{i}{h}^{\left(i-1\right)}\right)\:,$$

where $\:{W}_{e}^{i}$ is the weight matrix and $\:{h}^{\left(i-1\right)}$ is the output of the previous layer and $\:\varnothing\:$ is the ReLU activation function.

### Latent layer Architecture - BAE

**Fig. 6:**
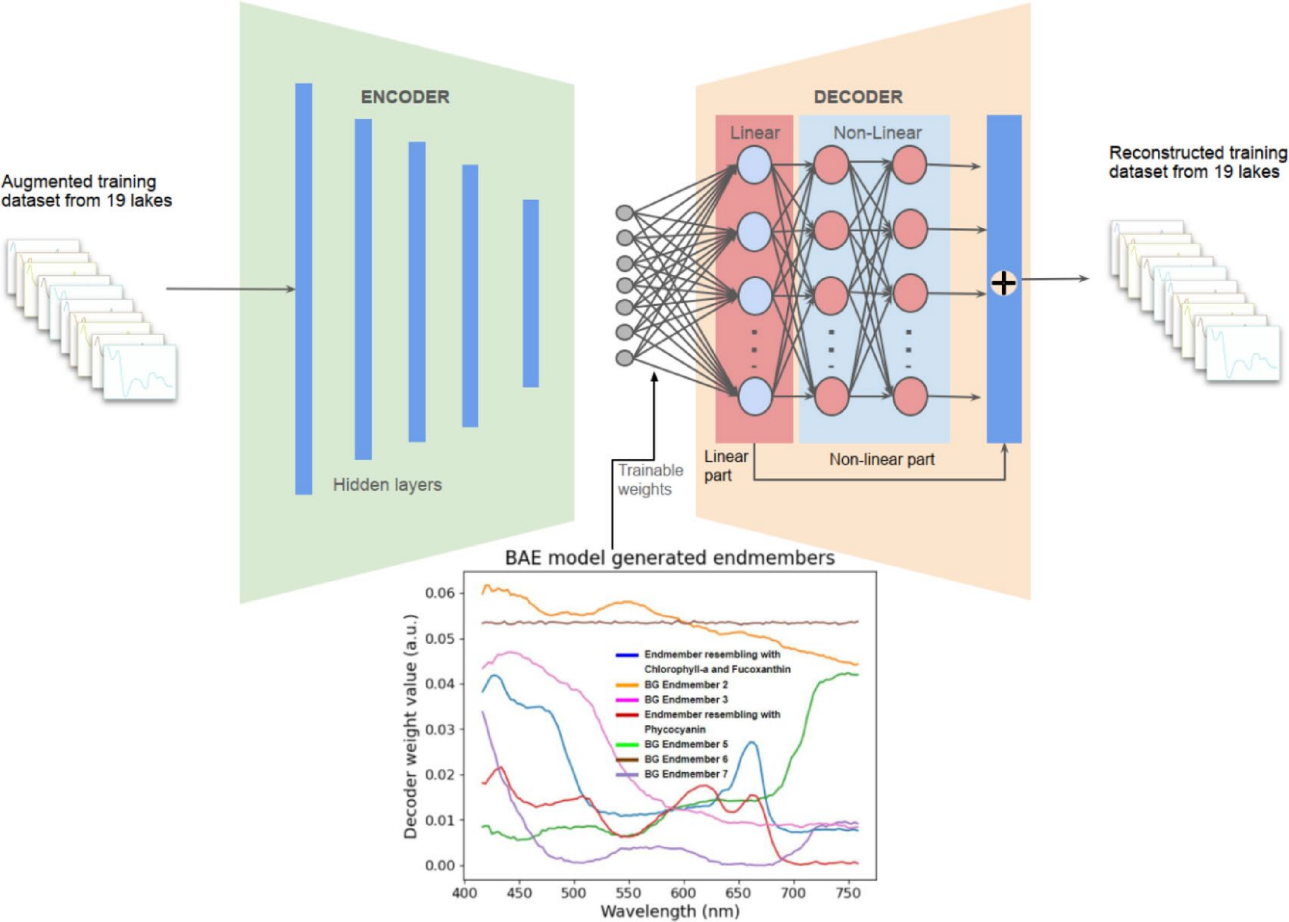
An illustration of the blind unmixing autoencoder (BAE) method. The autoencoder is trained on an augmented dataset of 19 lakes, with epochs varying based on the objective function and hyperparameter optimization. Post-training, latent layer activations provide abundance estimates, and the first layer decoder weights yield endmember estimates. The abundance estimates of the test set are derived by using only the encoder part and inputting the test dataset. The legend “BG” stands for background.

In BAE, the latent layer is represented with a latent vector $\:z$ is connected to the trainable weights of the decoder. (Fig. 6)

### Latent layer Architecture - EGAE

**Fig. 7:**
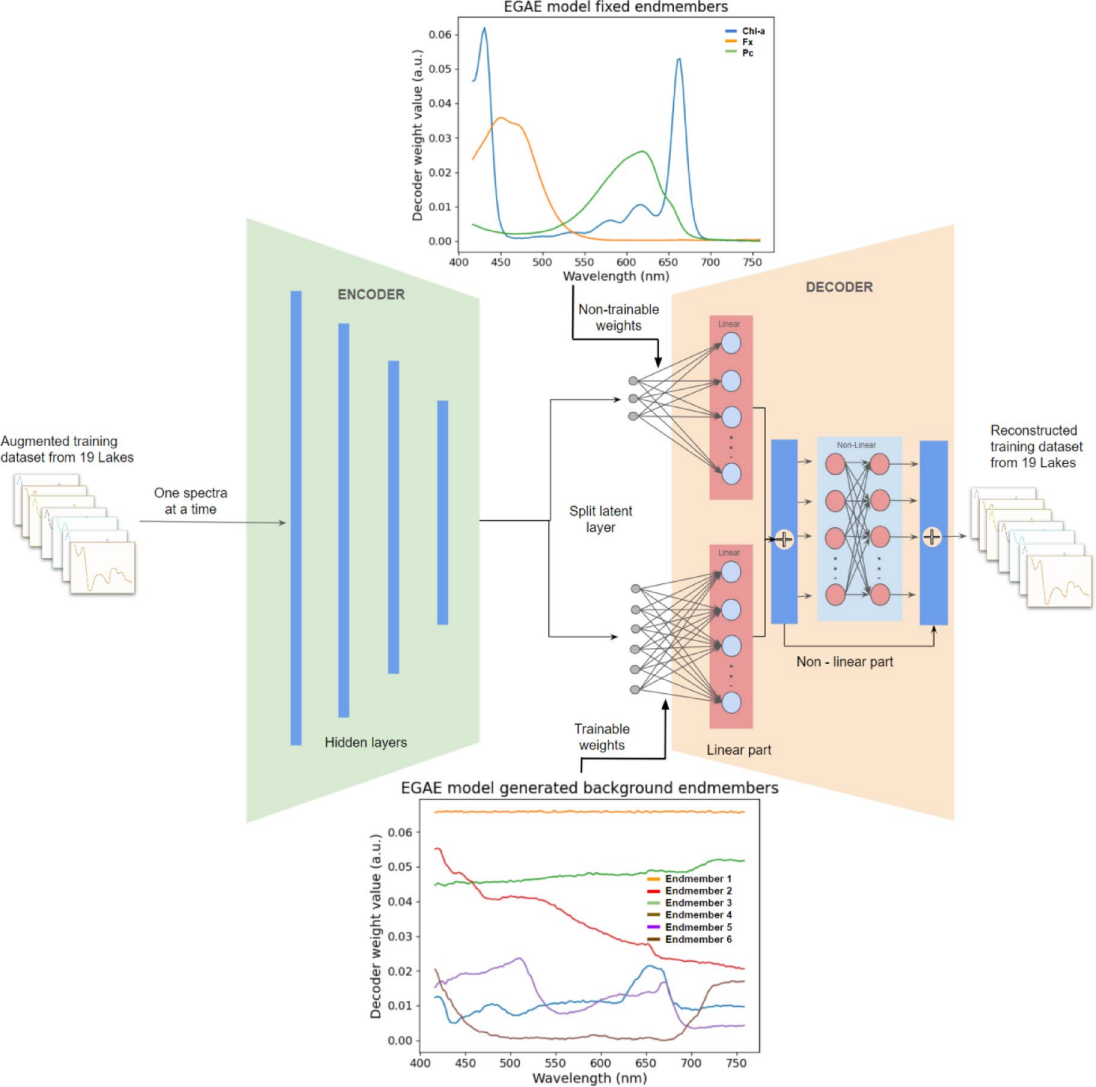
An illustration of the Endmember Guided unmixing autoencoder (EGAE) method. The autoencoder is trained on an augmented dataset of 19 lakes, with epochs varying based on the objective function and hyperparameter optimization. The latent layer is split into two segments: one connected to trainable weights and the other to non-trainable, frozen weights initialized with reference spectra of pigments, i.e. chl-a, fx^16^, and pc^17^. Post-training, latent layer activations provide abundance estimates, and the first layer decoder weights yield endmember estimates with the non-trainable weights capturing background endmembers labeled as Endmember 1 to 6. The abundance estimates of the test set are derived by using only the encoder part and inputting the test dataset.

The latent vector $\:z$ is split into two segments: the first segment $\:{z}_{f}$ is fully connected to a subsequent sublayer with frozen weights $\:{W}_{f}$derived from literature^16,17^ and the second segment $\:{z}_{nf}\:$is connected to a second sublayer with trainable weights $\:{W}_{nf}$ (Fig. 7). The latent vector can be defined $\:z\:=\:\left[{z}_{f},{z}_{nf}\right]$ where $\:z\in\:{\mathbb{R}}^{N\times\:R}$, $\:{z}_{f}\in\:{\mathbb{R}}^{N\times\:3}$ and $\:{z}_{nf}\in\:{\mathbb{R}}^{N\times\:R-3}$.

### Decoder - Linear component

The EGAE and BAE models differ in the architecture of their first decoder layer but share identical configurations in the subsequent layers.

### Decoder - Linear component - BAE

The decoder is designed to reconstruct the input with a linear structure followed by a non-linear structure. The output of this layer is

3 $$\:{o}^{1}=\:{\varnothing}\left({W}_{d}^{1}z\right)\:,$$

where $\:{W}_{d}^{1}\in\:{\mathbb{R}}^{h\times\:B}\:$ is the weight of the first layer of the decoder and $\:\varnothing\:$ is the ReLU activation function.

### Decoder - Linear component - EGAE

The first layer of the decoder of EGAE consists of two sublayers to separately process the frozen and non-frozen segments of the latent layer (Fig. 7). The output of this layer is

4 $$\:{o}^{1}\:=\:{\varnothing}\left({{W}_{d}^{1\left(f\right)}\:z}_{f}\:\right)+\:{\varnothing}\left({{W}_{d}^{1\left(nf\right)}\:z}_{nf}\:\right),$$

where $\:{W}_{d}^{1\left(f\right)}\in\:{\mathbb{R}}^{3\times\:B}$ and $\:{W}_{d}^{1\left(nf\right)}\in\:{\mathbb{R}}^{R-3\times\:B}$, with $\:\varnothing\:\:$as the activation function for both sublayers.

### Decoder Non-Linear component

In BAE and EGAE, the endmember non-negativity is addressed with a non-negative kernel constraint.

The output $\:{o}^{1}$of the first decoder layer is used as input of the non-linear component defined by a fully connected neural network without bias weights. This non-linear component represents the non-linear interaction among the endmembers weighted by associated abundances. Research shows that a neural network with two hidden layers can model arbitrary non-linear relationships among the inputs^12,22^. We employed two hidden layers to capture the nonlinear relationships among the endmembers, as higher-order photon interactions, although theoretically possible, tend to be weak in practical scenarios. The outputs of the decoder’s second layer

5 $$\:\:{y}_{1}=\:\:g\left({W}_{d}^{2}{o}^{1}\right)\:,$$

and the third layer

6 $$\:{y}_{2}=\:\:g\left({W}_{d}^{3}{y}_{1}\right)\:,$$

are activated using the Leaky ReLU activation function $\:g$.

The reconstructed spectrum

7 $$\:\widehat{x}\:=\:{o}^{1}\:+\:\phi\:\left({o}^{1}\right)$$

combines linear and non-linear outputs, where $\:{o}^{1}$ corresponding to Eq. 3 in BAE and Eq. 4 in EGAE (Figs. 5 and 6). The non-linear function $\:\phi\:\left({o}^{1}\right)$ represents the output of third layer $\:{y}_{2}$.

In comparison to Eq. (1), abundance vector $\:S$ corresponds to latent layer activations $\:z$. The endmember matrix M corresponds to weights $\:{W}_{d}^{1}$ in the case of BAE and vertical concatenation of the frozen and non-frozen weights $\:\left[{W}_{d}^{1\left(f\right)},{W}_{d}^{1\left(nf\right)}\right]$ in the case of the EGAE.

The models were trained on Nvidia Tesla V100-SXM2 16 GB GPU units.

### Training and experimental setting

During the data pre-processing phase, we created augmented spectral data consisting of 10,000 training and 5,000 test samples, each sample containing 120 spectral bands. Simultaneously, we acquired ground truth concentrations for each sample for three pigments (chl-a, fx, pc). These measurements were arranged into NumPy array of size 10,000 × 3 for the training set and 5,000 × 3 for the test set, with each column representing one pigment.

In the EGAE, we scaled each reference spectra to freeze the weights corresponding to the pigment endmember. Accurate scaling is crucial, as improper scaling could result in unsuccessful unmixing. The EGAE, an enhanced version of the BAE model, incorporates slight modifications in the latent and decoder layers. We, therefore, scaled the literature spectra based on where the endmember was obtained in the BAE model and matched the peaks. We also employed cubic spline interpolation to create 120-band values that match the bands of the input spectral signatures, as the literature endmembers had different band values and required us to select specific bands between 416 and 758 nm. The Batch size was set at 32.

### Hyperparameter optimization

Studies indicate that various factors, including the input dataset, influence the effectiveness of hyperparameter optimization methods in hyperspectral data and autoencoders. We evaluated optimization methods such as Random Search, Bayesian, and Hyperband on BAE and EGAE, with Hyperband proving the quickest and most effective for hyperparameter optimization. Additionally, in autoencoder-based unmixing models, network loss measures similarity between input and reconstructed output rather than unmixing performance, which instead assesses the quality of the decoder’s weights and the encoder’s latent codes, differing from traditional methods that focus on convergence on endmembers or abundances.

For the EGAE and BAE models, utilizing different loss functions, key hyperparameters include the number of encoder layers (ranging from 1 to 20 with step size 1), number of endmembers (4 to 16 with step size 1), learning rate (choices of 1e-4, 1e-3, or 1e-2). Each layer’s units are independently optimized within a range of 4 to 256 with step size 4. The tuning process, facilitated by the Hyperband tuner, aims to minimize the reconstruction loss over a maximum of 100 epochs, with the Hyperband’s factor set to 3 for efficient hyperparameter searching. Specifically, for the EGAE utilizing a combined MSE and SAD as loss function, an additional hyperparameter was the weighting factor $\:\alpha\:$ between MSE and SAD.

### Loss function

The choice of similarity measure significantly affects the performance of unmixing models, and the impact varies depending on the input dataset. Scale-sensitive similarity measures degrade unmixing performance in datasets with considerable spectral variability, while scale-invariant measures perform better by emphasizing shape differences rather than scale discrepancies^13^.

In our study, we examined two models, BAE and EGAE, to evaluate the impact of different loss functions on unmixing performance for our hyperspectral dataset. For the BAE model, we found that the scale-variant loss function provided superior unmixing performance compared to the scale-invariant function. In contrast, the EGAE achieved better results using the scale-invariant function for unmixing the fx endmember, while the scale-variant function performed well for unmixing chl-a and pc.

The Spectral Angle Distance (SAD) and Spectral Information Divergence (SID) are commonly used scale-invariant similarity measures in unmixing. The SAD is given by

8 $$\:{\mathcal{L}}_{SAD}\left({x}_{p},{\widehat{x}}_{p}\right)={\text{cos}}^{-1}\left(\frac{\langle{x}_{p},{\widehat{x}}_{p}\rangle}{{||{x}_{p}||}_{2}{||{\widehat{x}}_{p}||}_{2}}\right)\:,$$

where $\:{\widehat{x}}_{p}$ is the reconstructed spectrum and $\:{x}_{p}$ is the input spectrum. The SID is defined as

9 $$\:{\mathcal{L}}_{SID}\:\left({x}_{p},{\widehat{x}}_{p}\right)=\sum\:_{n=1}^{B}{p}_{n}{log}\left(\frac{{p}_{n}}{{q}_{n}}\right)\:+\:\sum\:_{n=1}^{B}{q}_{n}{log}\left(\frac{{q}_{n}}{{p}_{n}}\right)\:,$$

where

$$\:{p}_{n\:}=\:\frac{{x}_{p,n}}{\sum\:_{n=1}^{M}{x}_{p,n}}\:and\:{q}_{n\:}=\:\frac{{\widehat{x}}_{p,n}}{\sum\:_{n=1}^{M}{\widehat{x}}_{p,n}}\:.$$

The scale-variant measure, i.e., Mean Squared Error (MSE), is

10 $$\:{\mathcal{L}}_{MSE}\left({x}_{p},{\widehat{x}}_{p}\right)={||{x}_{p}-{\widehat{x}}_{p}||}_{2}^{2}.$$

We employed a combined loss function

11 $$\:{\mathcal{\:}\mathcal{L}}_{MSE\:+\:SAD}\left({x}_{p},{\widehat{x}}_{p}\right)=\:\alpha\:\:{\mathcal{L}}_{MSE}\left({x}_{p},{\widehat{x}}_{p}\right)\:+\left(1-\alpha\:\right){\mathcal{L}}_{SAD}\left({x}_{p},{\widehat{x}}_{p}\right)$$

to utilize the advantages of both scale-variant (MSE) and scale-invariant (SAD) similarity measures. The weighted sum of these two measures is determined by the $\:\alpha\:$.

### Regularization

Total Variation regularization was applied to the weights of the linear part of the decoder. This regularization term penalizes large variations between adjacent weight values of the weight vector to promote smoothness in the reconstructed spectra. The regularization is defined as

12 $$\:{R}_{EM}\left({W}_{d}^{1}\right)={||\nabla\:{W}_{d}^{1}||\:}_{1},$$

where $\:\nabla\:{W}_{d}^{1}$ represents the horizontal difference operator applied to the weight matrix $\:{W}_{d}^{1}$ of the first layer of the decoder.

Regularization using the $\:{L}_{2}$ was applied to the subsequent fully connected layers of the decoder responsible for non-linear unmixing. The regularization terms are defined as

13 $$\:{R}_{1}\left({W}_{d}^{2}\right)\text{}=\:{||{W}_{d}^{2}||}_{2}^{2}$$

and

14 $$\:{R}_{2}\left({W}_{d}^{3}\right)\text{}=\:{||{W}_{d}^{3}||}_{2}^{2}$$,

where the weights $\:{W}_{d}^{2}$ and $\:{W}_{d}^{3}$ of the second and third layers of the decoder.

The total loss function $\:{\mathcal{L}}_{Total}$ of our model is given by

15 $$\:{\mathcal{L}}_{Total}=\:{\mathcal{L}}_{RE}+\:{\lambda\:}_{TV}{R}_{EM}\:\:{+\:{\lambda\:}_{1}\text{R}}_{1}\:{+\:{\lambda\:}_{2}\text{R}}_{2}\:.\:\:\:\:\:\:\:\:\:\:\:\:\:\:\:\:\:\:\:\:\:\:\:\:\:\:\:\:\:\:\:\:\:\:\:\:\:\:\:\:\:\:$$

The reconstruction loss function $\:{\mathcal{L}}_{RE}$ of the autoencoder model can be any of Eqs. 8, 9, 10, or 11. The regularization term for endmembers is given by $\:{R}_{EM}$. Regularizing coefficients $\:{\lambda\:}_{1}$, $\:{\lambda\:}_{2}$ and $\:{\lambda\:}_{TV}\:$are hyperparameters that are chosen by the hyperparameter optimization model.

### Error metrics

The performance of the models in estimating abundance was measured using the Pearson correlation coefficient with respect to the ground truth assessment.

The stability of the models was assessed using the coefficient of variation, determined for the slope of the linear regression of the scatter plot of ground truth versus abundance estimates for each pigment. This evaluation was conducted for the 10 best-performing models for BAE using MSE as the loss function and for EGAE using MSE, SAD, SID, and combined MSE and SID as loss functions.

## Conclusion

The BAE model could unmix the absorbance spectrum of chl-a, fx and their abundance correlating strongly with measured concentrations but failed to unmix pc. The EGAE offered a higher correlation between the endmember abundances and the ground truth assessment of chl-a and fx and the capability to unmix the absorbance spectrum of the cyanobacteria pigment pc. The sensitivity of the BAE for the number of endmembers, the number of layers, and the number of units in the layer introduced significant variability in abundance estimates of the endmembers. In contrast, the EGAE achieved efficient spectral unmixing with less sensitivity to neural network architecture and the number of endmembers. The integration of the pc endmember did not affect the abundance estimates for chl-a and fx. This indicated that more fixed endmembers could be added for analysis with minimal impact on the abundance estimation of other endmembers and demonstrated the endmember-guided unmixing autoencoder model’s robustness and higher adaptability in complex spectral unmixing scenarios over the Blind unmixing model. The results of this study showed that spectral unmixing with the EGAE could be a powerful approach to estimating the abundance of various optical components in spectral data.

## Electronic supplementary material

Below is the link to the electronic supplementary material.

Supplementary Material 1

## Data Availability

The hyperspectral images are available via Fairdata IDA with DOI: https://doi.org/10.23729/20a62a08-e776-47f4-96e5-e8e3475d8c4a and the corresponding pigment data are available via the Environmental Information Data Centre (EIDC) with DOI: https://doi.org/10.5285/7794cbfa-1d74-4885-b303-8c20f4608728. The machine learning models and scripts/software will be published openly after publication of the manuscript and are available for reviewers upon request.
