# Supplementary material for: Blind and endmember guided autoencoder model for unmixing the absorbance spectra of phytoplankton pigments
Source: Sci Rep. 2025 Apr 16;15:13157. doi: 10.1038/s41598-025-96023-5 (PMC12003665; doi:10.1038/s41598-025-96023-5)
Supplement: Supplementary file 1 — Supplementary Material 1 [file 41598_2025_96023_MOESM1_ESM.docx]

**Supplementary material**

**Supplementary table 1.** The similarity between BAE - SID endmembers resembling reference endmembers in rad in the 8 best models from the training set. Mean SAD is the mean spectral angle distance between the unmixed endmembers and absorbance spectra derived from (Clementson, 2019; Suresh, 2009).

|  | **Models** | | | | | | | | **Mean SAD** |
| --- | --- | --- | --- | --- | --- | --- | --- | --- | --- |
|  | 1 | 2 | 3 | 4 | 5 | 6 | 7 | 8 |  |
| **chl- a** | 0.65 | 0.67 | 0.57 | 0.81 | 0.73 | 0.75 | 0.68 | 0.74 | 0.70 |
| **fx** | 0.52 | 0.42 | 0.40 | 0.34 | 0.45 | 0.5 | 0.36 | 0.49 | 0.44 |
| **pc** | 0.73 | 0.69 | 0.74 | 0.82 | 0.73 | 0.78 | 0.52 | 0.60 | 0.70 |

**
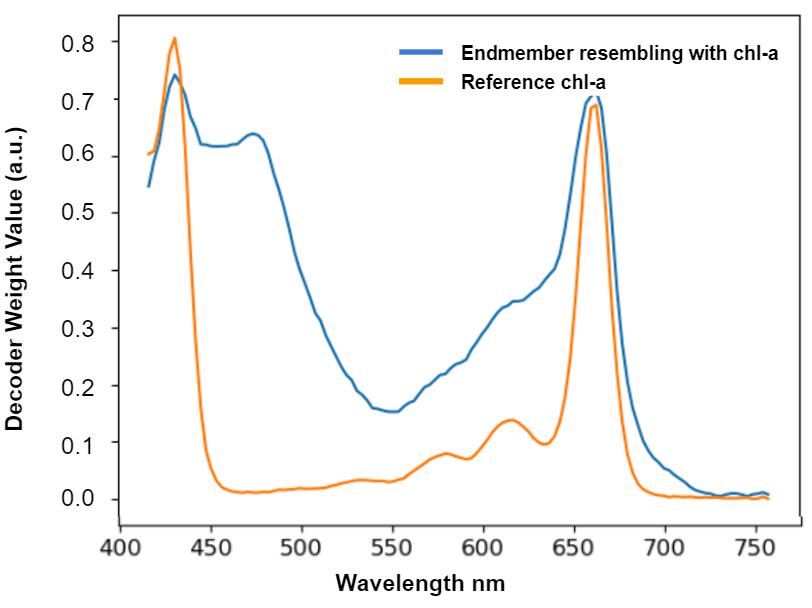

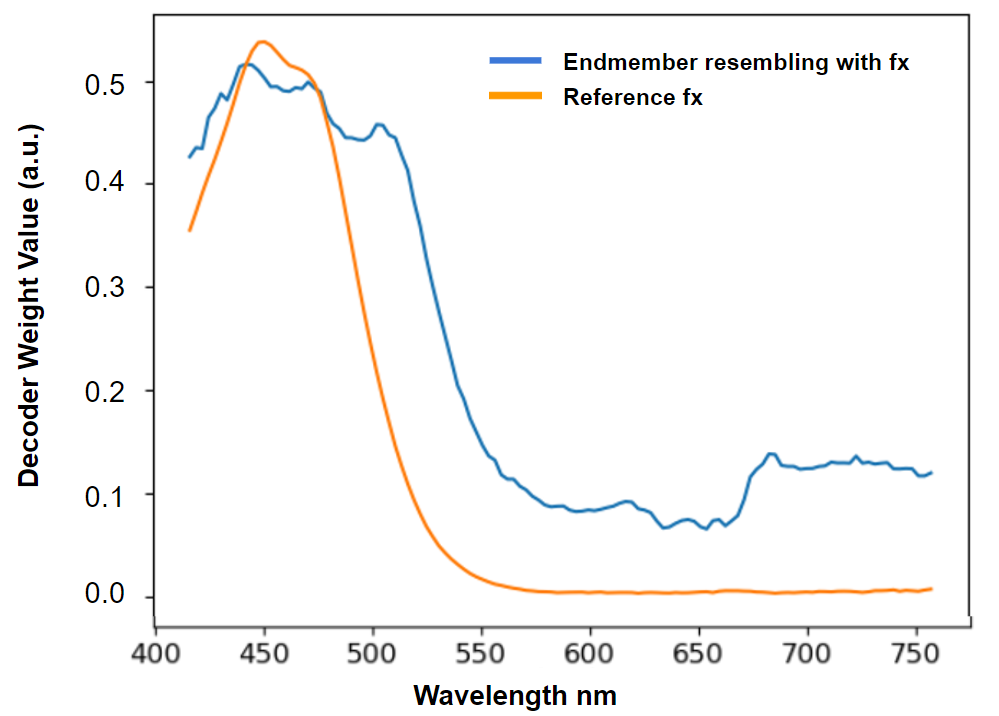

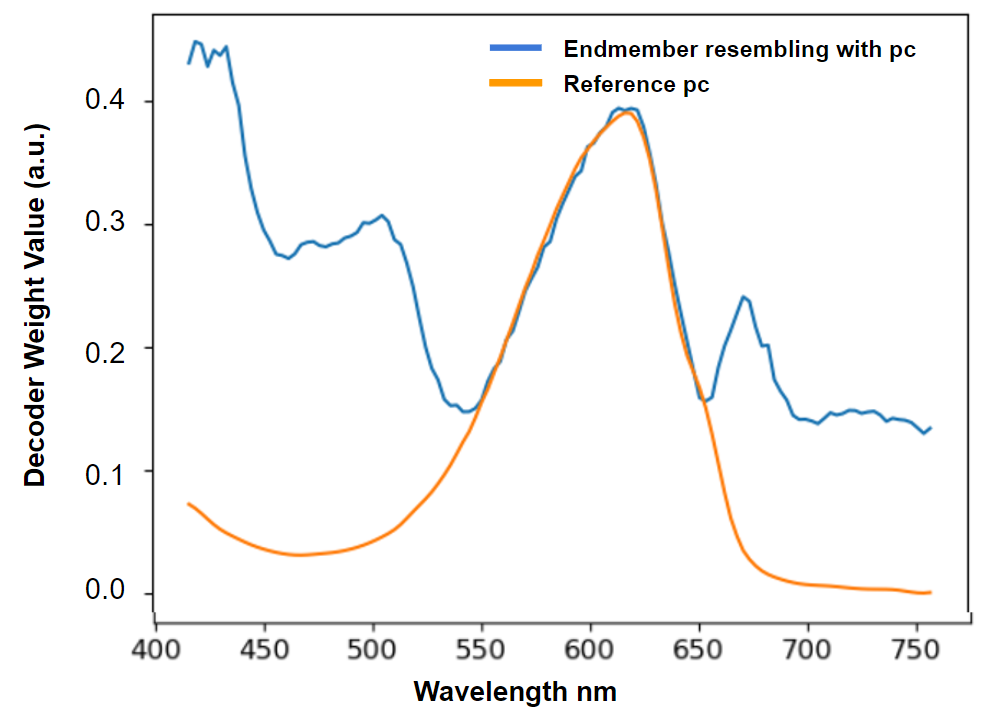
**

1. **
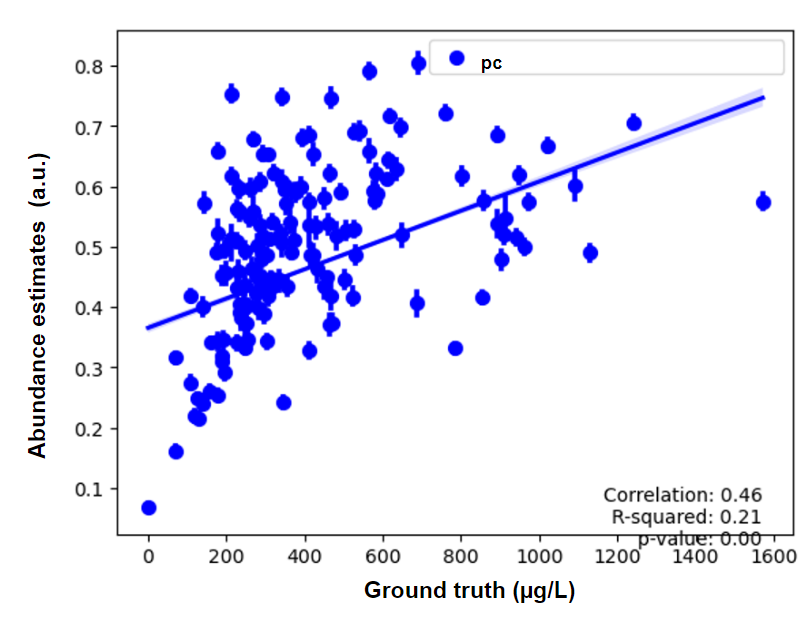
** (b) (c)


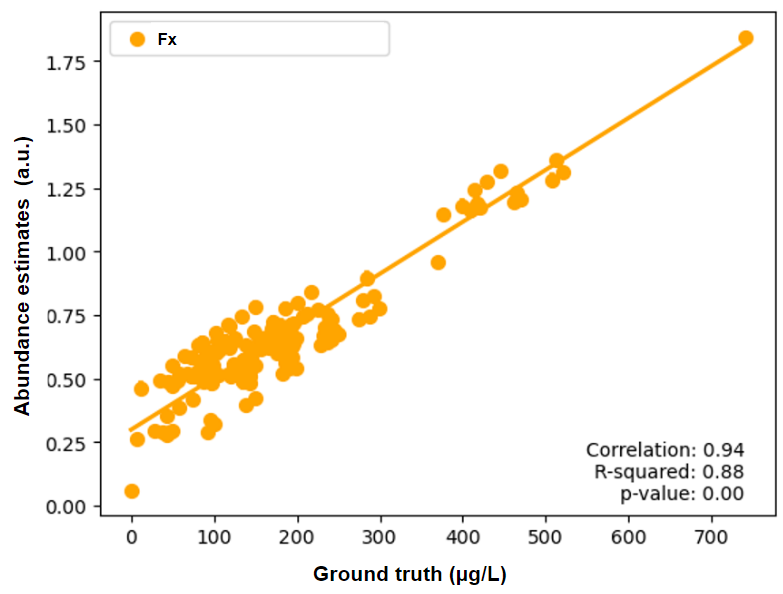
**
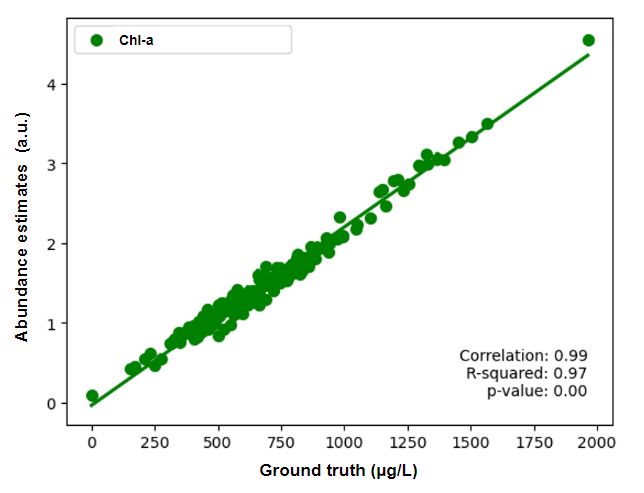
**

(d) (e) (f)


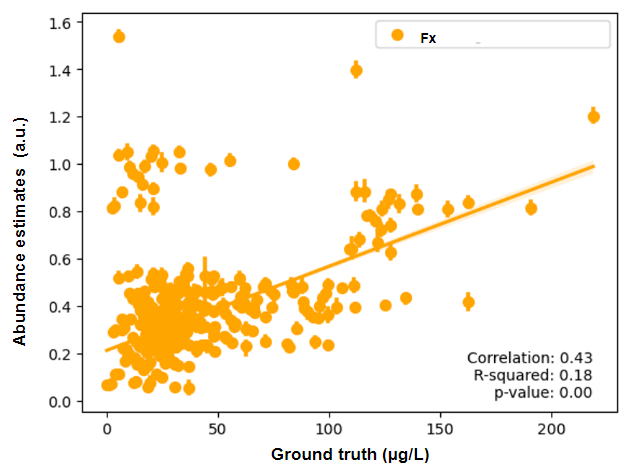
**
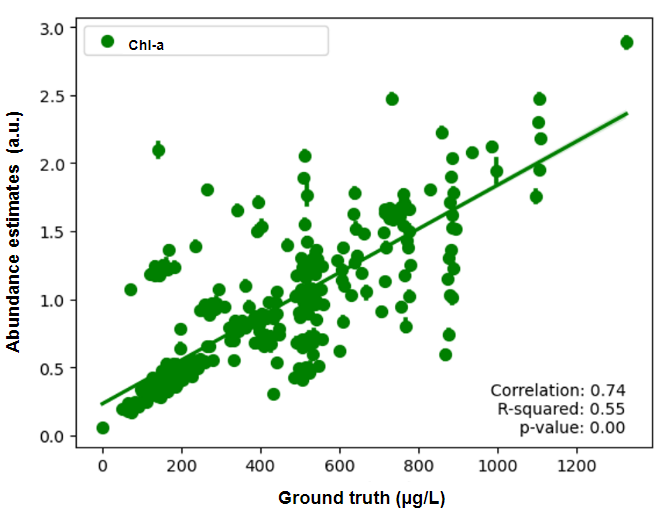
**

**
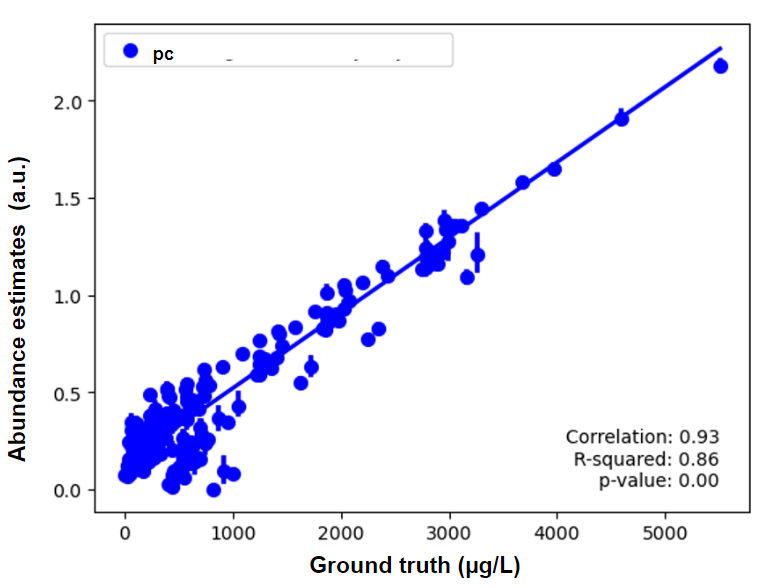
**

**(e) (f) (g)**

(g) (h) (i)

**Supplementary figure 1** Endmember estimates (a-c) and scatter plots between ground truth and abundance estimates for a selected BAE model with SID loss for the test (d-f) and training set (g-h). Scatter plots 1d and 1g show a strong positive correlation for chl-a (test and training), scatter plots 1e and 1h show a strong (test) and a moderate (training) positive correlation for fx, and scatter plots 1f and 1i show a moderate (test) and a strong positive (training) correlation for pc.


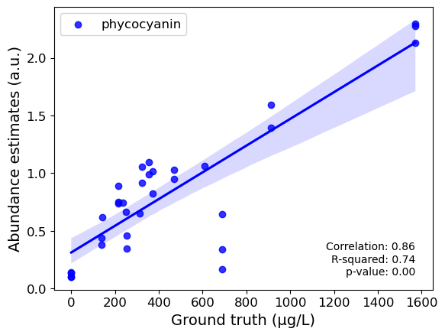

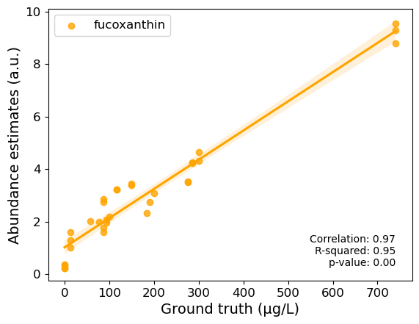

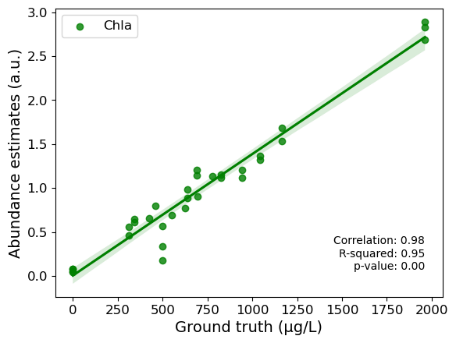


1. (b) (c)

**Supplementary figure 2** Scatterplots (2a, 2b, 2c) show a strong positive correlation for chl-a, fx and pc in non-augmented test data from Loch Leven. These results were similar to those of the augmented test data. All results are statistically significant (p<0.001).


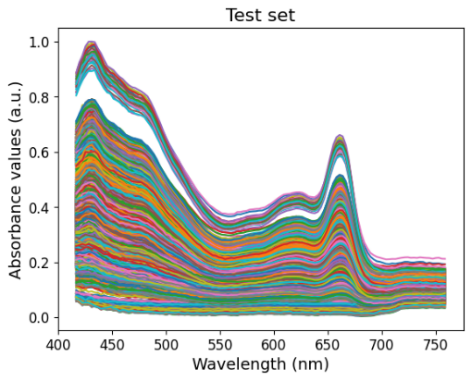

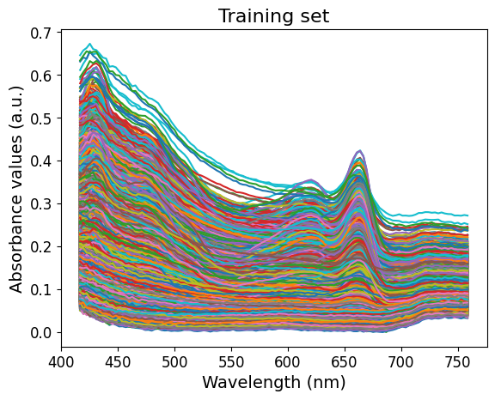


1. (b)


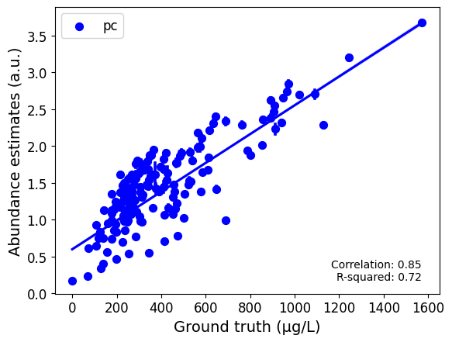

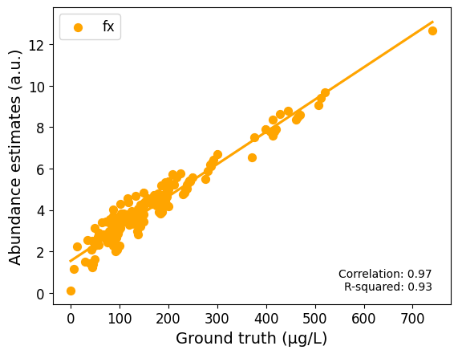

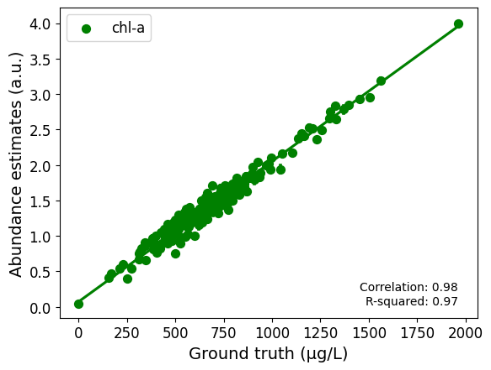


(c) (d) (e)


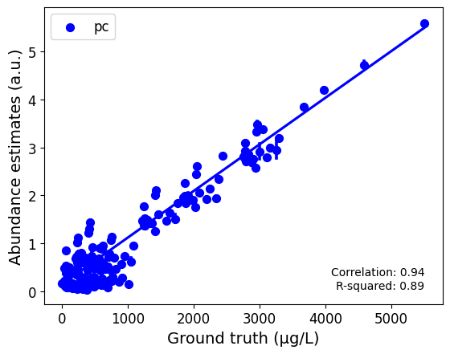

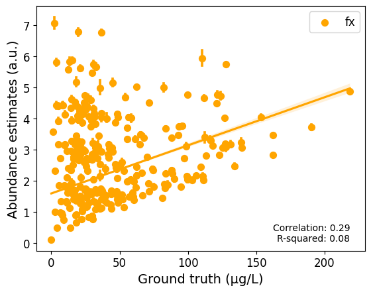

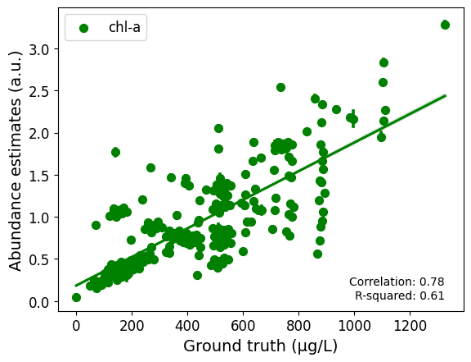


(f) (g) (h)

**Supplementary figure 3**: Training and test sets (a-b) without additional noise, and scatter plots of ground truth vs. abundance estimates for the selected EGAE model with MSE loss: test set (c-e) and training set (f-h). Abbreviations: chl-a (chlorophyll-a), fx (fucoxanthin), pc (phycocyanin). Correlation refers to Pearson correlation. All results are statistically significant (p<0.001).


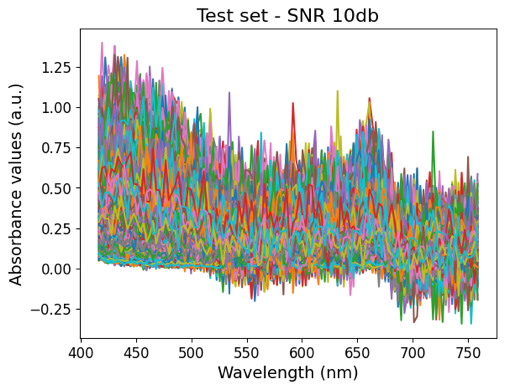


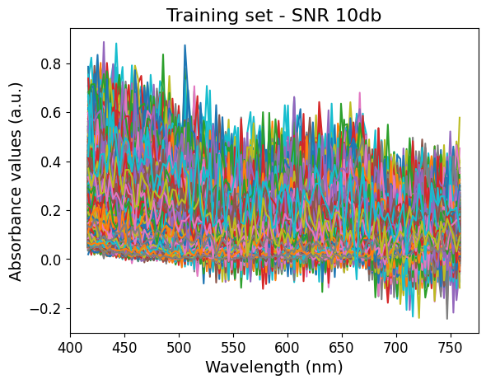


1. (b)


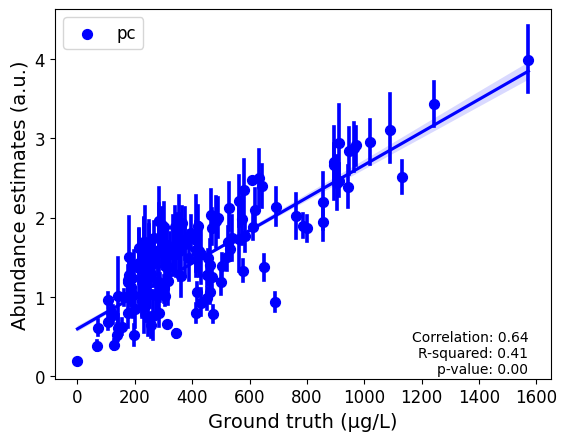

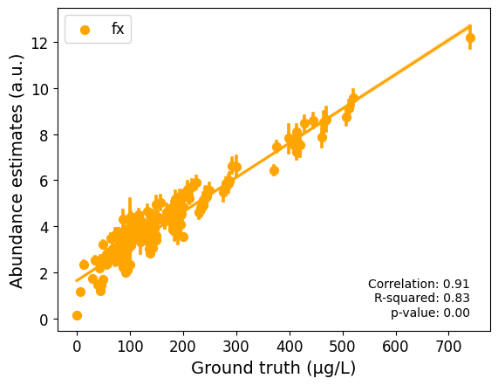

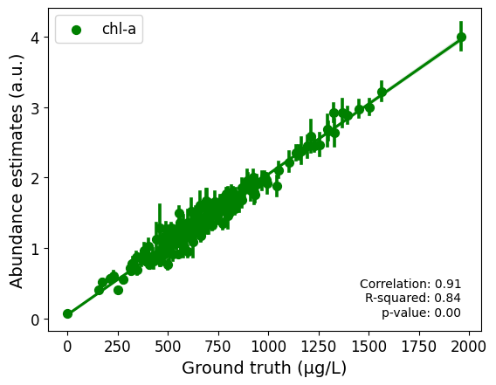


(c) (d) (e)


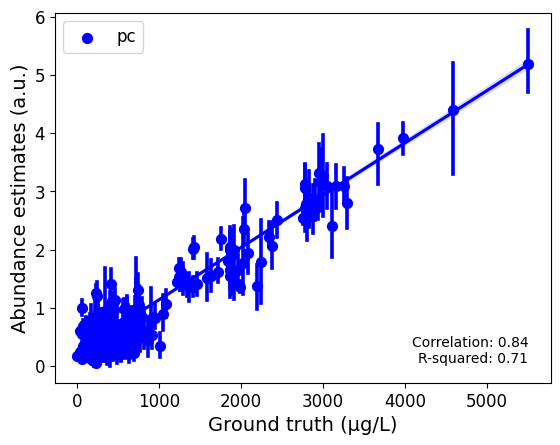

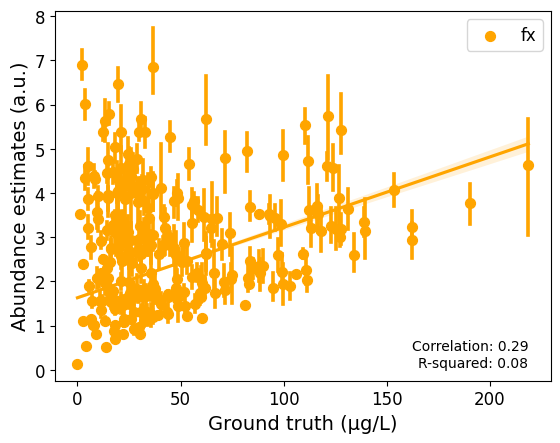

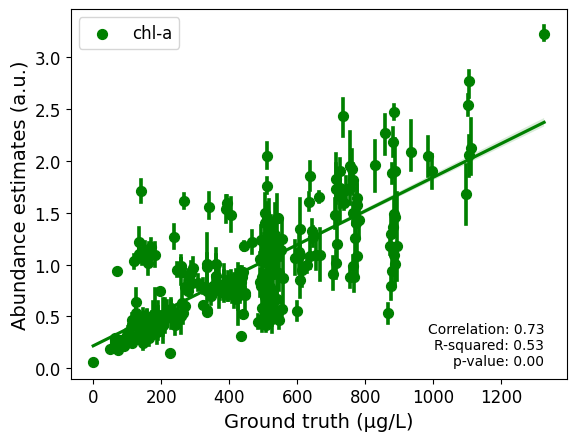


(f) (g) (h)

**Supplementary figure 4**: Training and test sets (a-b) with 10dB SNR, and scatter plots of ground truth vs. abundance estimates for the selected EGAE model with MSE loss: test set (c-e) and training set (f-h). Abbreviations: chl-a (chlorophyll-a), fx (fucoxanthin), pc (phycocyanin). Correlation refers to Pearson correlation. All results are statistically significant (p<0.001).

**Supplementary table 2:** Pearson correlation between ground truth and abundance estimates across various SNR levels for the selected EGAE model with MSE loss on test and training sets. The model was chosen using Hyperband optimization. Abbreviations: chl-a (chlorophyll-a), fx (fucoxanthin), pc (phycocyanin). All results are statistically significant (p < 0.001).

| SNR level (dB) | Test set | | | Training set | | |
| --- | --- | --- | --- | --- | --- | --- |
|  | Chl-a | Fx | Pc | Chl-a | Fx | Pc |
| 10 | 0.91 | 0.91 | 0.64 | 0.73 | 0.29 | 0.84 |
| 20 | 0.97 | 0.96 | 0.81 | 0.77 | 0.29 | 0.93 |
| 30 | 0.98 | 0.97 | 0.84 | 0.78 | 0.29 | 0.94 |
| 40 | 0.98 | 0.97 | 0.85 | 0.78 | 0.29 | 0.94 |


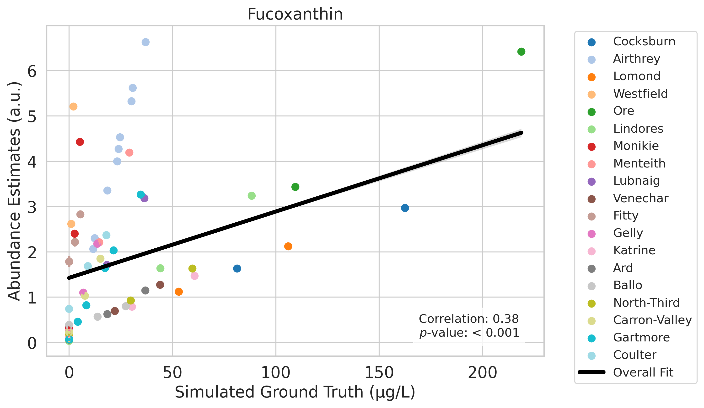

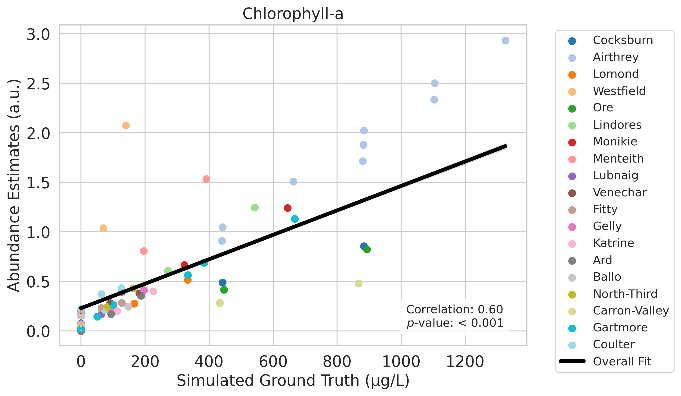


1. (b)


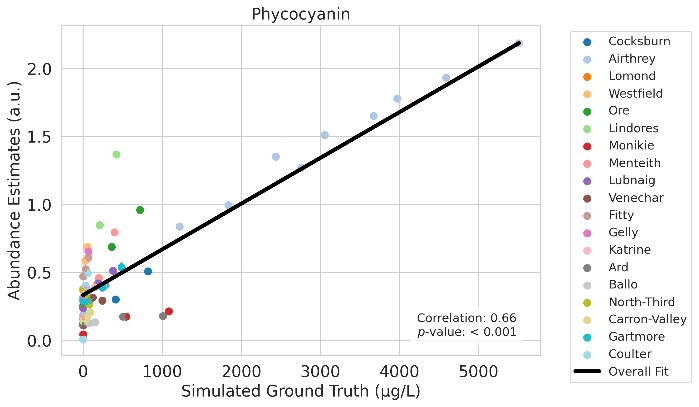


(c)

**Supplementary figure 5:** Scatter plots for (a) chl-a, (b) fx, and (c) pc showing leave-one-out results of simulated ground truth versus abundance estimates derived from testing with the omitted lake. In these plots, Loch Leven was part of the training but not the testing, as its results have already been shown separately. All results are statistically significant (p<0.001).
